# Novel Thiazolidin-4-ones as Potential Non-Nucleoside Inhibitors of HIV-1 Reverse Transcriptase

**DOI:** 10.3390/molecules24213821

**Published:** 2019-10-23

**Authors:** Anthi Petrou, Phaedra Eleftheriou, Athina Geronikaki, Melpomeni G. Akrivou, Ioannis Vizirianakis

**Affiliations:** 1 Department of Pharmaceutical Chemistry, School of Pharmacy, Aristotle University of Thessaloniki, Thessaloniki 54124, Greece; anthi.petrou.thessaloniki1@gmail.com; 2 Department of Biomedical Sciences, School of Health Sciences, International Hellenic University, Nea Moudania 57001, Greece; eleftheriouphaedra@gmail.com; 3 Department of Pharmacology and Pharmacognosy, School of Pharmacy, Aristotle University of Thessaloniki, Thessaloniki 54124, Greece; akrivoumg@pharm.auth.gr (M.G.A.); vizir@pharm.auth.gr (I.V.)

**Keywords:** thiazolidin-4-ones, NNRTIs, AIDS, HIV-1 reverse transcriptase, molecular docking

## Abstract

Background: HIV is the causative agent of Acquired Immunodeficiency Syndrome (AIDS), an infectious disease with increasing incidence worldwide. Non-nucleoside reverse transcriptase inhibitors (NNRTIs) play an important role in the treatment of AIDS. Although, many compounds are already being used as anti-HIV drugs, research for the development of new inhibitors continues as the virus develops resistant strains. Methods: The best features of available NNRTIs were taken into account for the design of novel inhibitors. PASS (Prediction of activity spectra for substances) prediction program and molecular docking studies for the selection of designed compounds were used for the synthesis. Compounds were synthesized using conventional and microwave irradiation methods and HIV RT inhibitory action was evaluated by colorimetric photometric immunoassay. Results: The evaluation of HIV-1 RT inhibitory activity revealed that seven compounds have significantly lower ΙC_50_ values than nevirapine (0.3 μΜ). It was observed that the activity of compounds depends not only on the nature of substituent and it position in benzothiazole ring but also on the nature and position of substituents in benzene ring. Conclusion: Twenty four of the tested compounds exhibited inhibitory action lower than 4 μΜ. Seven of them showed better activity than nevirapine, while three of the compounds exhibited IC_50_ values lower than 5 nM. Two compounds 9 and 10 exhibited very good inhibitory activity with IC50 1 nM.

## 1. Introduction

Acute Immunodeficiency Syndrome (AIDS) is a result of the infection and destruction of T-lymphocytes by the human immunodeficiency virus (HIV). Over 60 million people are infected with the virus placing AIDS as the fourth cause of death worldwide [1,2,3].

Non-nucleoside reverse transcriptase inhibitors, as a part of Highly Active Antiretroviral Therapy (HAART), remain in the first line of fighting against AIDS. However, research for the development of novel NNRT inhibitors still continues as the virus develops resistant strains, limiting their use.

Among the approved by FDA compounds are several heterocyclic compounds such as benzoxazin-2-one (etravirine), dipirydo [1,4] diazepine-6-one (etravirin) [4], piperazine and indolyl (delaverdine) moieties [5,6]. Except of these drugs many other molecules have been found to display RT inhibitory action [7,8,9,10,11,12,13], among which are: benzothiazine dioxides [14], N1,N3-disubstituted uracils [15], 6-arylmethyl-substituted S-DABOs [16], adamantyl-substituted thiazolidine-4-ones [17,18] and many others [19,20,21,22,23,24,25,26,27]. The 2,3-diaryl-thiazolidin-4-one scaffold appeared as a selective NNRTI [28,29]. Modeling studies on the HIV-1 RT inhibitory activity of 2,3-diaryl-thiazolidin-4-ones showed the importance of overall hydrophobicity of the analogues and the presence of a heteroaryl system over the 3-aryl moiety for better HIV-1 RT inhibitory activity [30,31,32,33,34].

Computational methods are an important part of the drug design process and they are presently used to get a deeper understanding into the drug-enzyme interactions.

In this study, taking into account the best features of available NNRTIs a large number of novel 2,3-diaryl-thiazolidin-4ones were designed. The most promising compounds were selected with the aid of Molecular docking studies and prediction of spectra of biological activity for compounds by computer program PASS, synthesized and evaluated.

## 2. Results and Discussion

### 2.1. Rational Design of the Compounds

It is known that HIV-1 reverse transcriptase (HIV-1 RT) has a flexible structure that contains two binding domains. The active site characterized by the catalytic triad of Asp110, Asp185 and Asp186 residues, where the deoxynucleoside triphosphates are bound by the physiological process, and an allosteric site of NNRTIs about 10 Å away from the active site.

The NNRTIs binding pocket is hydrophobic, as most of its amino acids are hydrophobic (Pro59, Leu100, Val106, Val179, Leu234 and Pro236) and five of them are aromatic (Tyr181, Tyr188, Phe227, Trp229 and Tyr232). Certain hydrophilic amino acids such as Lys101, Lys103, Ser105, Asp132 and Glu224 are also present [35]. For the binding of NNRTIs to the reverse transcriptase enzyme, their ability to adopt a configuration characterized as a “butterfly orientation” is needed [36]. Aromatic rings are usually present in both wings of “batterfly” conformation. A heeterocyclic ring or other hydrogen donor/acceptor groups stabilized the complex via hydrogen bond formation, usually with Lys101.

**Figure 1:**
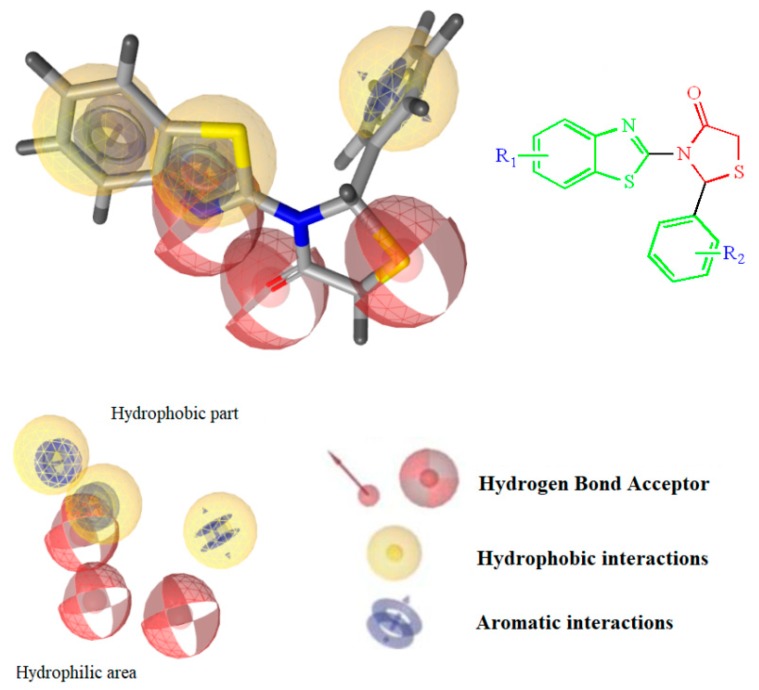
Structure and Basic Characteristics of the Designed Compounds.

The design of the compounds was based on the presence of the above characteristics and consist of a benzothiazole ring connected via thiazolidinone moiety with a phenyl ring. The benzothiazole and the phenyl ring serve as the wings of the ‘’butterfly’’ confirmation, forming hydrothobic interactions, while the thiazolidinone moiety involves in the formation of hydrogen bond interactions especially with Lys101 (Figure 1). Different substituents at the benzothiazole and phenyl rings were used to determinate the compounds with the best predicted activity.

### 2.2. Computational Prediction of Anti-HIV Activity

PASS prediction of anti-HIV RT inhibitory activity was performed for several dozen designed molecules, from which thirty two were selected for synthesis and testing. Prediction was carried out using PASS online [37] version. For all selected compounds, anti-HIV inhibitory activity was predicted with Pa values in range of 0.297–0.704 (Table 1). The calculated Pa values for most of compounds were less than 0.5, indicating their relative novelty compared to the structures of the compounds from the PASS training set [38,39].

### 2.3. Molecular Docking Prediction

**Figure 2:**
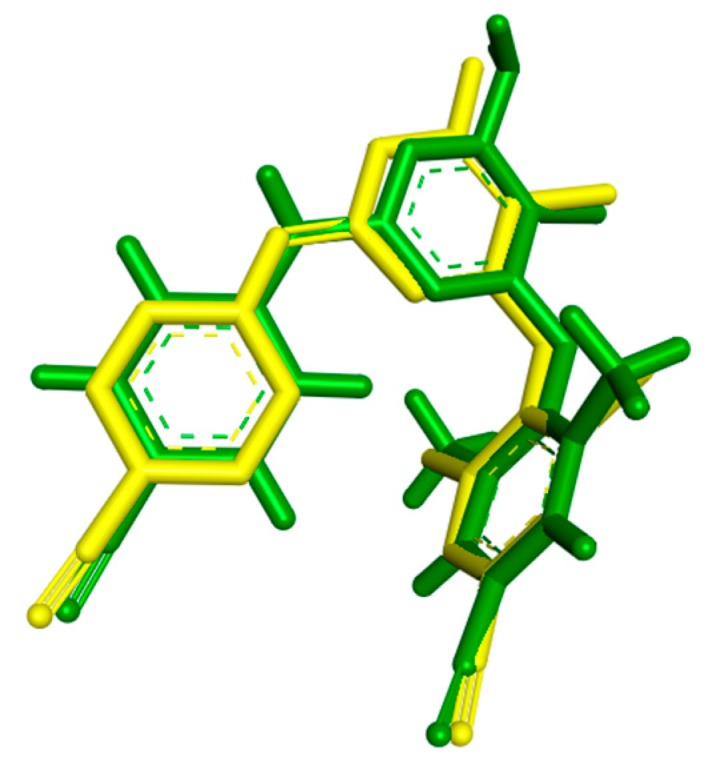
Conformation of TMC 125-crystal Structure (green) as Compared to the Docked Conformation of TMC 125 (yellow).

For the docking studies the enzyme of RT in complex with the inhibitor TMC 125, the most widely used in the literature, was selected (PDB code: 3MEC). At first stage, a validation test was performed to certify the program’s reliability. Firstly, the ligand TMC 125 (etravirine), in the conformation found in the crystal structure, was extracted and docked back into the corresponding binding pocket to determine the ability of Autodock to reproduce the orientation and position of the inhibitor observed in the crystal structure. According to docking results, the orientation of the docked TMC 125 inhibitor was very close to that found in the crystal structure (Figure 2). The low RMS deviation of 0.58 Å between the docked and the crystal ligand is an indication of the very good alignment of the experimental and calculated positions.

All designed compounds and the reference drug nevirapine were docked to the HIV-1 reverse transcriptase enzyme using the same parameters (Table 1). Both the (S) and (R) isomer of each compound were docked separately. It was observed that the (R) isomer exhibited proportionaly decreased free binding energy for each compound [40,41,42].

**Figure 3:**
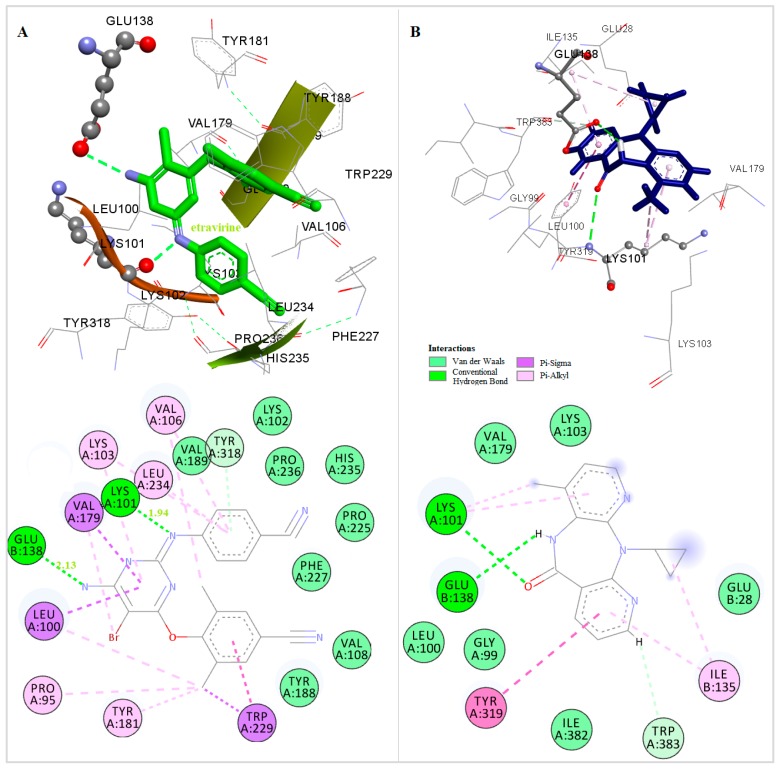
Docked Conformation of TMC125(etravirine) (**A**) and Nevirapine (**B**) in the Allosteric Center of HIV-1 RT.

The three-dimensional docked structures of the studied compounds were compared with the X-ray crystallographic structure of TMC 125 (Figure 3). Detailed analysis of the binding mode found in the docked compounds-RT and other NNRTIs-RT crystal complexes showed that the most of the compounds could bind to RT in a butterfly-like conformation. Like TMC 125 (Figure 3), most of the compounds are involved in the formation of a hydrogen bond with Lys101, which was observed in the X-ray crystallographic structure of the TMC 125-RT complex [43].

From the 132 compounds designed, the **32** showed very good binding scores and taking into account also the prediction results by PASS, were chosen for the synthesis (Table 1 and Table 2).

**Figure 4:**
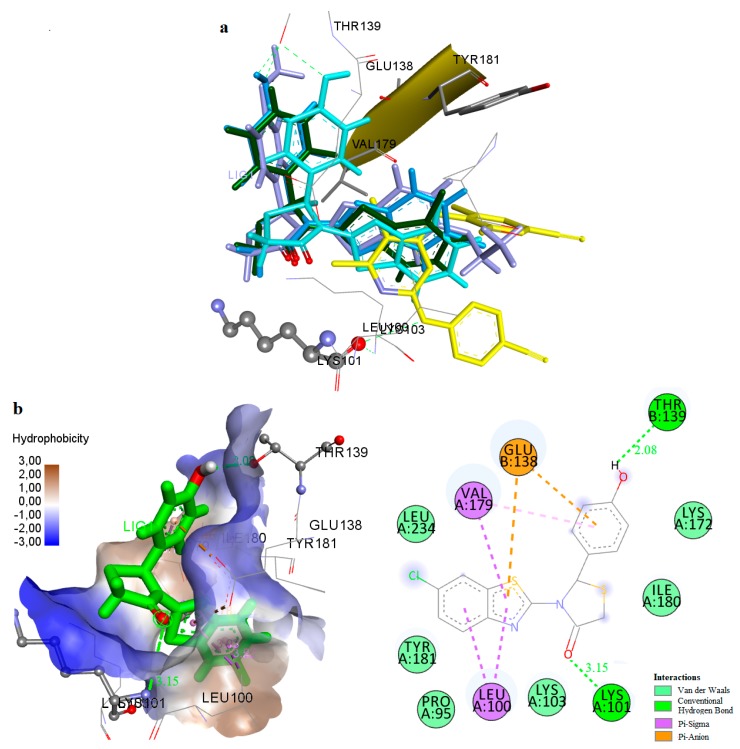
(**a**) Docking of TMC125 (yellow) and Compounds **1** (green), **9** (blue), **14** (light blue) and **27** (purple) to the Allosteric Site of HIV-1 RT. (**b**) Docking of Compound **9** (green) in the Binding Site of HIV-1RT.

It was observed that these compounds bind to the allosteric center of the enzyme occupying a similar configuration to TMC 125 inhibitor (Figure 4a, best docked conformations of compounds **1**, **9**, **14**, **27**). Comparison of the X-ray structure of TMC 125 with compound **9** (−14.63 kcal/mol) (Figure 4b), showed that this compound occupies similar configuration to TMC 125 inhibitor and forms a hydrogen bond with Lys101. Specifically, the CH_2_-S moiety of 4-thiazolidinone is oriented towards a channel formed between the residues Lys101, Val179 (p66 subunit) and Glu138 (p51 subunit). The nitrogen atom of Lys101 interacts with the oxygen atom of the 4-thiazolidinone forming a hydrogen bond. The substituted benzothiazole ring interacts hydrophobically with the residues Leu100, Val179, Glu138 and Leu234. These hydrophobic interactions contribute significantly to the stabilization of the compound-RT complex. As shown in Figure 4b, the 4-hydroxyphenyl ring is located inside the cavity surrounded by the residues Pro95, Thr139, Glu138, and Ile180. It is important to mention that the presence of the hydrogen bond with Lys101 is characteristic of many NNRTIs inhibitors. This phenomenon is also observed in the crystallographic TMC 125-RT and 9-Cl TIBO-RT complexes [43].

Compounds with higher docking scores exhibit a different binding mode from those with lower docking scores. In this type of binding, the substituted phenyl ring is oriented to a “channel” formed by Leu100, Glu138, Ile180 and Val179. This orientation results in the CH_2_-S moiety of 4-thiazolidinone being removed away from Lys101 and thus, being not able to interact and form a hydrogen bond with it. This fact contributes significantly to the ability of these compounds to form a stable complex with the enzyme and, potentially, to their intended action.

### 2.4. Prediction of Toxicity

According to Lasar model throughout OpenTox, all the compounds found to be at the category IV with LD_50_ between 500 mg/kg and 1000 mg/kg and they are saved for use (Table 3).

### 2.5. Chemistry

**Scheme 1:**
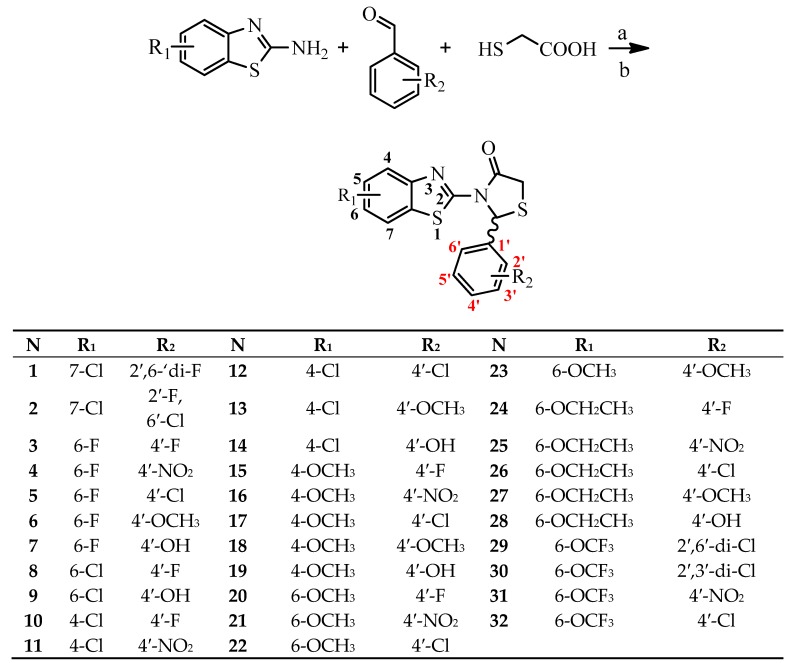
General synthetic procedure. Reagents and conditions: (a) Conventional method: toluene, reflux for 18–32 h, (b) microwave-assisted technic: 100 °C, power 100 W, 30 min.

Synthesis of the compounds was performed by one pot method according to Scheme 1 [44] and for some compounds by microwave assisted one-pot procedures (Scheme 1). According to conventional method, the mixture of the suitable aminobenzothiazole, substituted benzaldehyde and mercaptoacetic acid was refluxed in toluene for 18–32 h. All products were obtained as racemates in low yield. Instead using microwave assisted one-pot synthetic procedure the reaction time was reduced to 30 min. The final products were obtained in good yield. All synthesized compounds were characterized by TLC and spectroscopic methods (IR, ^1^H NMR, ^13^C-NMR and MS for some compounds).

In IR spectra stretching absorption bands at 1700 cm^−1^ (strong) of C=O, 1600 and 1540 cm^−1^ of -C–C- and 3200 of –OH were detected. In ^1^H-NMR spectra signal at 8.10–6.89 ppm as well as at 4.35–4.10 ppm are attributed to aromatic protons and proton of the position 2 of thiazolidinone moiety respectively. The rest of the protons appeared at the expected chemical shifts. In ^13^C-NMR spectra peaks were observed for C=O group at δ 172–170 ppm, for C-2 of benzothiazole ring at δ 161–165 ppm and for C-2 and C-5 of thiazolidinone moiety at 53–60 ppm and at 30–34 ppm respectively (see experimental).

### 2.6. HIV-1 RT Inhibitory Action

The results of the evaluation of RT inhibitory activity are presented in Table 4. All compounds at the concentration of 4 μM exhibited inhibitory action varying from 21% to 83%.

Seven compounds were found to have significantly lower IC_50_ than nevirapine (0.3 μM), the best of which displayed IC_50_ value lower than 1μM. The order of the compound’s potency can be presented as follows: **10** ≥ **9** > **2** > **14** > **3** > **1** > **8** > nevirapine > **20** > **27** > **13** > **28** > **15** > **16** > **23** > **26** > **17** > **19** > **18** > **22** > **4** > **7** = **12** = **21**. Nine compounds, the **5**, **6**, **11**, **24**, **25**, **29**, **30**, **31**, **32**, exhibited IC_50_ values greater than 4 μM.

The study of structure-activity relationships revealed that the activity of compounds depends not only on the nature of substituent and its position in benzothiazole ring but also on the nature and position of substituents in benzene ring. The presence of 6-Cl substitution in the benzothiazole moiety in combination with the presence of hydroxyl group at position 4′ of the benzene ring (**9**) appeared to be beneficial for the HIV RT inhibitory action. The same effect was observed in case of 4-Cl substitution in the benzothiazole moiety combined with the presence of 4′-F group in benzene ring (**10**).

The replacement of 4′-F substitution in compound **10**, one of the two most active compounds, by 4′-OH (**14**) remarkably decreased the activity although compound **14** still remained a very active compound being more active than nevirapine. On the other hand, compound **2** with the presence of 7-Cl substituent in benzothiazole moiety and 2′-F, 6′-Cl (**2**) substitution in benzene ring was the third most active compound. The replacement of 6′-Cl (compound **2**) by 6′-F in benzene ring (**1**) significantly decreased activity compared to compound **2**. While introduction of 6-F in benzothiazole moiety and 4′-F substituent in benzene ring (**3**) increased the activity compared to compound **1**, the replacement of 6-F by 6-Cl led to compound **8** with decreased activity in comparison with compounds **1** and **3**. However, despite that compounds **1**, **3** and **8** were less active compared to compounds **9**, **10**, **2** and **14** they still appeared to be more active than the reference compound.

The replacement of 4′-F substituent by nitro-, chloro-, methoxy and hydroxyl group respectively resulted to compounds with decreased activity (**4**–**7**). The 6-F benzothiazole substituted derivatives **4**–**7** showed IC_50_ values ≥ 4 μM. On the contrary, the 6-Cl benzothiazole substituted derivatives **8** and **9** exhibited good inhibitory action with compound **9** being one of the most active compounds. It seems that in this group of compounds the presence of the hydrogen donor 4′-OH group is beneficial (**9**). In case of the 4-Cl benzothiazole substituted derivatives (**10**–**14**) it appeared that the presence of the hydrogen acceptor 4′-F group was the most beneficial (**10**) followed by the hydrogen donor 4′-OH (**14**) and 4′-OMe group (**13**). The rest of compounds exhibited IC_50_ values ≥ 4 μM. It is interesting to mention that 4-OCH_3_ as well as 6-OCH_3_ benzothiazole substituted derivatives showed activity with IC_50_ values between 1.5 and 3 μM, showing small differentiation in concern to phenyl substituents. An exception was observed only for the 4′-nitrophenyl derivatives with 6-methoxy group in the benzothiazole moiety (**21**, IC_50_ 4 μM) being the less active compound of the group. In general, 4′-nitro substitution in benzene ring was detrimental for all groups of compounds with exception of the derivatives with methoxy group in benzothiazole moiety.

The derivatives **26**–**28** (4′-Cl, 4′-OMe, 4′-OH) with 6-ethoxy group in benzothiazole moiety displayed activity almost similar to that of 6-methoxy substitution (R_1_) with IC_50_ 1.93–2.48 μM. Regarding the 4′-fluoro- and 4′-nitrophenyl derivatives (**24**, **25**) IC_50_ values higher than 4 μM were observed.

In conclusion, it is interesting to notice that the presence of chlorine in position 4′ (R_2_) of benzene ring was negative for all derivatives with halogen substituted benzothiazole moiety independent of its position. The presence of a chloro- substituent at the benzothiazole ring (R_1_) at positions **4**, **6** or **7** significantly increases the inhibitory effect (**1**, **2**, **8**, **9**, **10**, **14**). On the other hand, the introduction of -OCF_3_ substituent into the benzothiazole system further reduces the activity as compared to the chloro-substituted derivatives, with most of the compounds of this group (**30**–**32**) displaying IC_50_ values higher than 4 μM.

### 2.7. Docking Analysis

**Figure 5:**
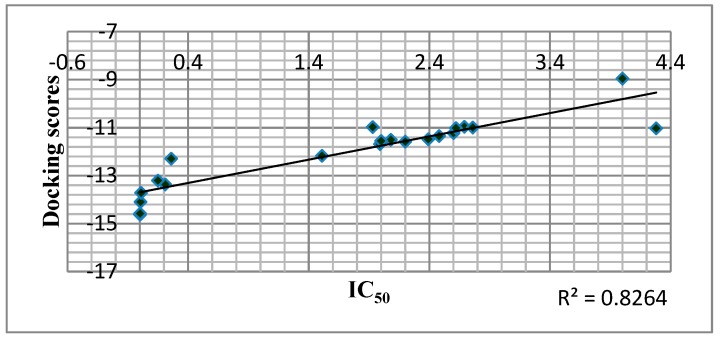
Correlation of Predicted Free Binding Energy of the Compounds in the Allosteric Center of the Enzyme with IC_50_ of the Compounds.

Docking analysis effectively predicted RT inhibitory action in most cases as shown in Figure 5.

**Figure 6:**
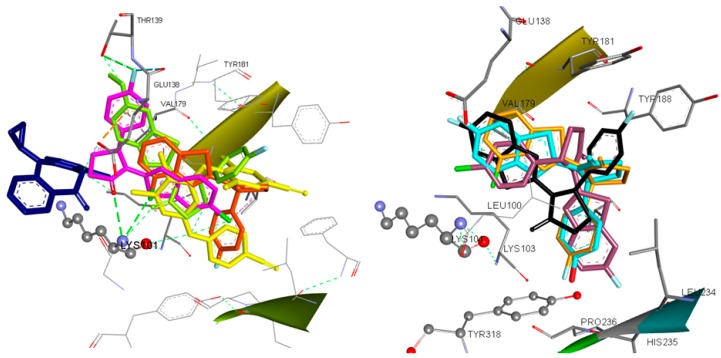
(Left) Docking of Etravirine (yellow), Nevirapine (blue) and Compounds **8** (magenta), **20** (light green) and **10** (orange) in the Binding Site of HIV-1RT. (Right) Docked Conformations of Compounds **3** (black), **5** (dark purple), **7** (light blue) and **10** (orange) in the Binding Site of HIV-1RT.

According to docking results, most of the compounds are placed within the NNRTIs binding pocket between the place equipped by the classic butterfly shaped compound, nevirapine, and the binding site of etravirine or within the etravirine binding pocket (Figure 6).

Interestingly, the most active compounds structurally belong to three different groups and adopt three different orientations.

**Figure 7:**
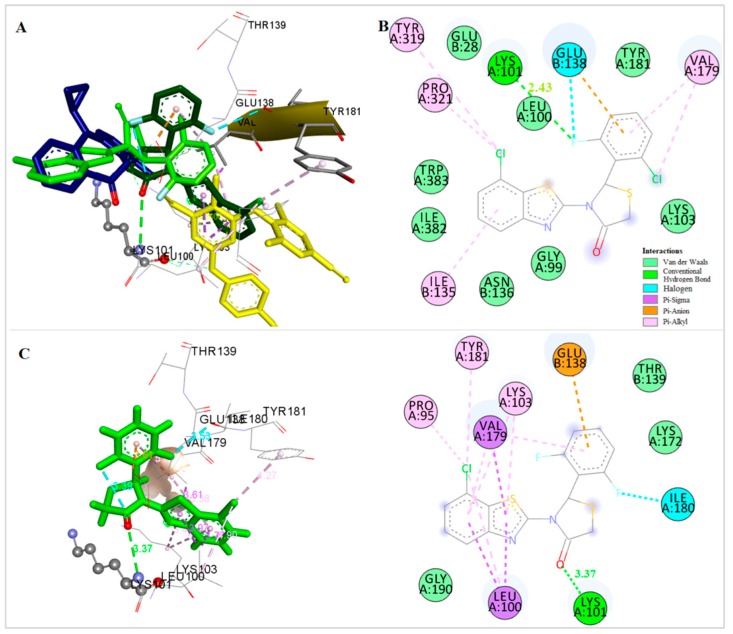
(**A**) Docking of Compounds **1** (dark green), **2** (light green), Nevirapine (dark blue) and Etravirine (yellow) at the Allosteric Site of HIV-1 RT. (**B**) Docking of Compound **2** at the Allosteric Site of HIV-1 RT. (**C**) Docking of Compound **1** at the Allosteric Site of HIV-1 RT.

An orientation most close to the nevirapine binding place is adopted by compound **2**, the 2′-F,6′-Cl derivative of the subgroup of compounds bearing a Cl substituent at the 7-position of the benzothiazolyl moiety (Figure 7A,B). More precisely, the benzothiazole and thiazolidinone moieties occupy positions similar with the “wings” of nevirapine. The benzothiazolyl moiety is placed, in vicinity to the amino acids Trp383 and Ile135 with which it forms pi interactions. The thiazolidinone ring is placed in the area of Lys103, while the phenyl ring is placed near Glu138, Tyr181 and Lys101, forming pi interactions with Glu138. Halogen and hydrogen bonds interactions are also formed between the F atom and the amino acids Glu138 and Lys101, respectively, stabilizing the complex.

A different orientation is adapted by the 2′,6′-di-fluoro derivative of the same subgroup, compound 1 (Figure 7A,C). In this case, the thiazolidinone ring is placed in vicinity to Lys 101, which forms a hydrogen bond with the CO group of this moiety. The benzothiazolyl group is placed in the same area where the central aromatic ring of etravirine is bound, towards Tyr181, Leu100 and Val179 with which it participates in pi interactions. The two fluoro-atoms at the o-positions of the phenyl moiety participate in halogen bond interactions, an intramolecular one with CO of the thiazolidinone ring stabilizing the conformation of the molecule and a second one with Ile180.

**Figure 8:**
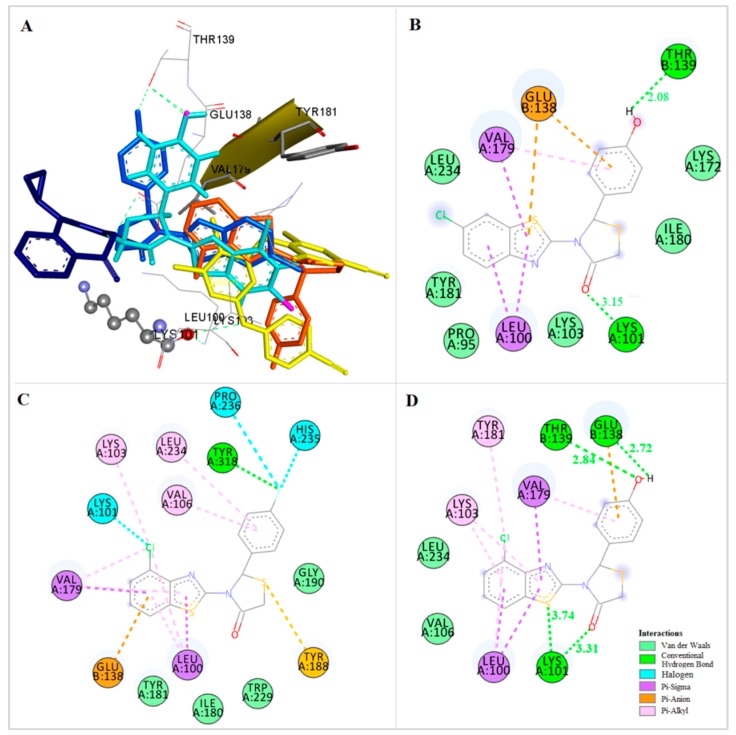
(**A**) Docking of compounds **8** (magenta), **9** (light blue), **10** (orange), **14** (cyan), nevirapine (dark blue) and etravirine (yellow) at the allosteric site of HIV-1 RT. (**B**) Docking of compound **9** at the allosteric site of HIV-1 RT. (**C**) Docking of compound **10** at the allosteric site of HIV-1 RT. (**D**) Docking of compound **14** at the allosteric site of HIV-1 RT.

A similar orientation is adopted by several active compounds like compounds **8**, **9**, the two more active compounds of the subgroup bearing a Cl-atom at position 6 of the benzothiazolyl moiety (R_1_) (Figure 8A,B,D) and compound **14**, the most active compound of the subgroup bearing a Cl-atom at the 4-position of the benzothiazolyl moiety (Figure 8A,D). Among these, compounds **9** and **14** belong in the four more potent out of the thirty-two tested compounds with IC_50_ values at the nanomolar range. In case of compounds **9** and **14**, a hydrogen bond is formed between the CO group of the thiazolidinone ring and Lys101 while a second hydrogen bond is formed between hydrogen acceptor substituents of the 4-position of the phenyl ring and Thr139. An additional hydrogen bond between the H-atom of the 4′-OH substituent and the Glu138 is also formed in compound **14**. Pi interactions between the rings of the benzothiazolyl moieties and the amino acids Leu100 and Val179 were observed in all these cases.

**Figure 9:**
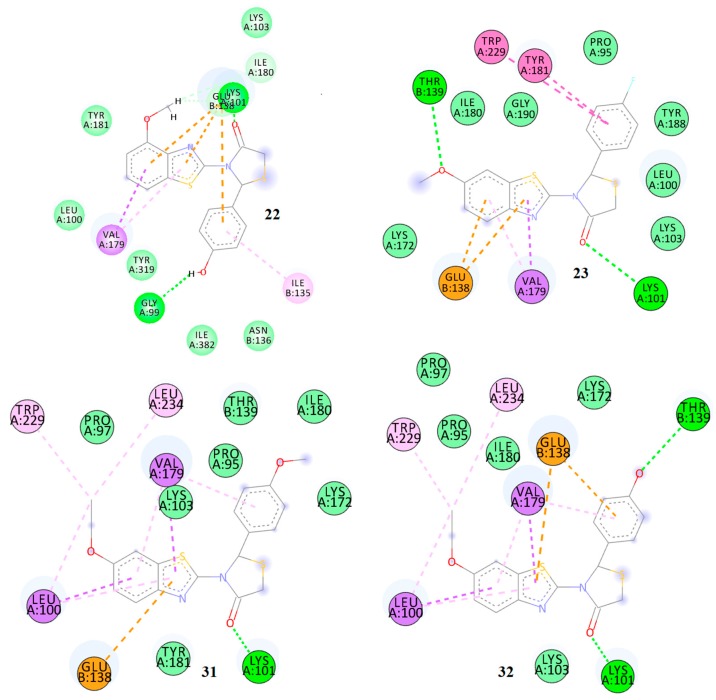
2D docking diagrams of compounds **19**, **20**, **27**, and **28** at the allosteric site of HIV-1 RT.

An orientation enabling pi interactions between the aromatic rings of the benzothiazolyl moiety and the amino acids Leu100 and Val179, as well as hydrogen bond formation between Lys101 and the CO group of the thiazolidinone ring were also observed in the active compounds of the 4-OMe, 6-OMe and 6-OEt subgroups such as compounds **18**, **20**, **27** and **28** (Figure 9).

On the contrary, the presence of the strongly electro-negative 4′-F (R_2_) substituent at compound **10** (Figure 8A) leads to a totally different orientation placing the compound with the phenyl moiety in vicinity to Tyr318 with which the F-atom forms a hydrogen bond and close to Pro236 and His235 forming halogen bonds. The differentiation of this compound from the other derivatives and most known NNRTIs where hydrogen bond interactions with Lys101 play a crucial role in complex stabilization is an interesting characteristic. However, the benzothiazolyl moiety is still placed in the area of Lys101, Val179 and Leu100 forming pi interactions with the last two amino acids (Figure 8A,C). In this conformation, the phenyl and the thiazolidinone moieties occupy the places occupied by the edge aromatic rings of etravirine while the benzothiazolyl moiety is placed in the area of the central ring.

Similar orientations, with the phenyl or benzothiazole moiety placed towards Tyr 318 are also observed in other cases (Figure 6).

In general, the presence of a Cl substituent at the 7-, 6- or 4- position of the benzothiazolyl moiety facilitates an orientation which enables complex stabilization via pi-interactions and hydrogen bond formation involving either the CO group of the thiazolidinone ring or hydrogen acceptor/donor substituents of the phenyl ring. In case of subgroups bearing hydrogen acceptor substituents at the benzothiazole moiety, involvement of these substituents in hydrogen bond formation also favors complex stabilization such as in case of compound **3** of the 6F-subgroup, **18** of the 4-OMe subgroup, **23** of the -OEt subgroup and **33** of the -CF_3_ subgroup.

### 2.8. Cytotoxicity Assessment

**Figure 10:**
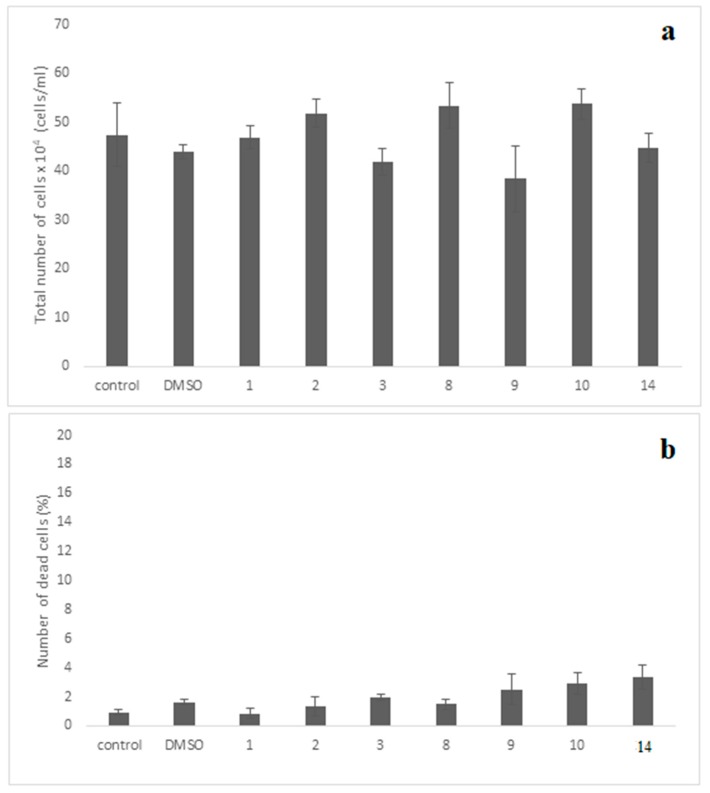
Assessment cell growth (**a**) and cell death (**b**) of MRC-5 cells exposed to various compounds. The MRC-5 cell cultures were incubated separately with each of the compounds at 10^−5^ M (10μΜ) for 48 h. The cell growth was determined by measuring the cell number in cultures under the microscope using the Neubauer counting chamber, as described in the “Materials and Methods”. Moreover, the dead cells accumulated in these cultures were also evaluated using the Trypan-blue exclusion-dye method, as presented in “Materials and Methods”. The results shown above indicate the mean numbers ± SD of two independent biological experiments. The diagrams shown above and the t-test statistical analysis were carried out using the GraphPad Prism 6.0 program. Notably, no statistical significance between the control-untreated culture with each one of compounds-treated cultures is seen (*p* values > 0.05).

For the evaluation of cellular cytotoxicity of the compounds, the MRC-5 normal cells were incubated for 48 h separately with each compound at the concentration of 10 μΜ. This concentration is much higher than IC_50_ values of all tested compounds (IC_50_ 0.001 (compounds **9**, **10**) and 0.01 (compound **14**). The evaluation revealed that compounds exhibited no evidence of cytotoxicity against this normal cell line employed, since no statistically significant change was observed with the cell growth to be ≥80% as compared to control-untreated culture (Figure 10a). Importantly, no significant dead cells accumulation was observed in the treated cultures, since their maximum number did not exceed that of 3.3% (Figure 10b).

## 3. Materials and Methods

### 3.1. Computer Simulation Methods

For docking calculations, the software AutoDock 4.2 was used [45]. The free energy of binding (ΔG) of HIV-1 Reverse Transcriptase complex with the tested compounds was generated using this molecular docking program.

The crystal structure of HIV-1 Reverse Transcriptase in Complex with TMC125 (PDB code: 3MEC) was downloaded from the Protein Data Bank [46]. For RT preparation, water molecules were deleted, polar hydrogens were added and Kollman United Atom charges were assigned. For the preparation of ligand structures, 2D structure was sketched in chemdraw12.0 software (Chemical Structure Drawing Standard; Perkin Elmer Informatics, Waltham, MA, USA) and converted to three-dimensional structures, mol2 format, for each ligand. Hydrogens were added to the structures and used for docking. The Grid center was calculated: 9.7037 12.7492 16.914 (xyz-coordinates), for HIV-1 Reverse Transcriptase. The grid size was set to 110  ×  110  ×  110 xyz points with grid spacing of 0.375 Å. All parameters used in docking were default. A primary blind docking was performed in order to discriminate the preferential binding sites of the ligand to the receptor. The ligand TMC125, as it is binded in the crystal structure was extracted and docked back into the analogous binding pocket to determine the ability of Autodock to replicate the position and orientation of the inhibitor in the crystal structure. Control docking showed that Autodock was able to determine the orientation, position and the same interactions of the crystallographic TMC125. The number of docking runs was 100. After docking, the 100 solutions were clustered into groups with RMS lower than 1.0 Ε. The resulting poses and interactions were visualized in Discovery studio 4.1 silent.

### 3.2. PASS Prediction

For the prediction of activity spectra of the designed compounds the program PASS was used. PASS, as already described in our paper [47] predicts the biological activity spectrum of a compound based on the analysis of structure–activity relationships of more than 1 million of known compounds. These compounds possess over eight thousand biological activities. The average accuracy of prediction is about 96%.

### 3.3. Prediction of Toxicity

For the prediction of toxicity of the designed compounds the Lazar model over the Open Tox Predict Program was used. OpenTox is a free online program, that provides access to experimental toxicity data, in Silico models (including (Q)SAR), and validation/reporting procedures. Lazar model predicts the toxicity of the compounds based on their structure by searching for similar compounds. Lazar is a k-nearest-neighbor approach to predict chemical endpoints from a training set based on structural fragments. It uses a SMILES file and precomputed fragments with occurrences as well as target class information for each compound as training input. It also features regression; in which case the target activities consist of continuous values. Lazar uses activity-specific similarity (i.e., each fragment contributes with its significance for the target activity) that is the basis for predictions and confidence index for every single prediction [48,49].

### 3.4. Chemistry

#### 3.4.1. General Procedure for the Synthesis of Thiazolidinones by Conventional Method

The appropriate heteroaromatic amine (1.0 mmol) and substituted benzaldehyde (1.2 mmol) were stirred in dry toluene under reflux condition followed by addition of mercatoacetic acid (2.0 mmol). The reaction mixture was refluxed for 18–32 h and then concentrated to dryness under reduce pressure. The residue was diluted in ethyl acetate and the organic layer was washed with 5% aq citric acid, water and 5% aq sodium hydrogen carbonate. The organic layer was dried over sodium sulfate and concentrated under reduced pressure to give a clear product. All the synthesized compounds were characterized by TLC, ^1^H-NMR, ^13^C-NMR and MS.

#### 3.4.2. Microwave Irradiation Experiments

All microwave irradiation experiments were carried out in CEM-Discover monomode microwave device, operating at a frequency of 2.45 GHz. Substituted aminobenzothiazole, equimolar amount of substituted benzaldehyde (1.5 mmol) and mercaptoacetic acid in absolut ethanol (3 mL) were placed in a 10 mL reaction vial containing a stirring bar. The vial was sealed with a Teflon septum and placed into the microwave cavity. It was irradiated at 100 °C using 100 W as maximum power for 30 min. at the end of the reaction the mixture was rapidly cooled with gas jet cooling to room temperature. The clean product was obtained after filter under reduced pressure.

#### 3.4.3. Synthesis of 3-(7-Chloro-benzo[d]thiazol-2-yl)-2-(2,6-difluorophenyl)thiazolidin-4-one (**1**)

Yield: 39%, m.p. 200–202 °C, R_f_: 0.66 (petroleum ether/ethyl acetate 8:2). IR (Nujol), cm^−1^: 1705 (C=O), 1541, 1102. ^1^H-NMR (500 MHz, CHCl_3_-d): δ = 3.86 (d, *J* = 16.38 Hz, 1H, CH_2_), 4.10 (d, *J* = 16.63 Hz, 1H, CH_2_), 6.83 (d, *J* = 18.59 Hz, 1H, N-CH-S), 7.21 (t, *J* = 8.56 Hz, 2H, Ar-C18, C20), 7.06–7.15 (m, 1H, Ar-C5), 7.61–7.72 (m, 3H, Ar-C5, C6, C19), 8.05 (d, *J* = 8.02 Hz, 1H, Ar-C4). ^13^C-NMR (500 MHz, CHCl_3_-d): δ = 33.12, 60.42, 113.05, 119.34, 123.02, 124.15, 125.03, 126.11, 126.79, 129.01, 130.00, 135.65 150.42, 162.16, 164.17, 171.12. MS: *m*/*z* = 382 (M^+^, 100%), 353 (33.3%), 372 (36%), 363 (28%), 348 (21%), 242 (72%).

#### 3.4.4. Synthesis of 3-(7-Chloro-benzo[d]thiazol-2-yl)-2-(2-chloro-6-fluorophenyl)thiazolidin-4-one (**2**)

Yield: 48%, m.p. 159–160 °C, R_f_: 0.52 (petroleum ether/ethyl acetate 8:2). IR (Nujol), cm^−1^: 1708 (C=O), 1541, 1101, 721. ^1^H-NMR (500 MHz, CHCl_3_-d): δ = 3.85 (d, *J* = 16.38 Hz, 1H, CH_2_), 4.12 (d, *J* = 16.63 Hz, 1H, CH_2_), 6.71 (d, *J* = 18.59 Hz, 1H, N-CH-S), 7.07 (t, *J* = 8.56 Hz, 1H, Ar-C19), 7.30–7.55 (m, 4H, Ar-C5, C-6, C18, C19), 8.06 (d, *J* = 8.02 Hz, 1H, Ar-C4). ^13^C-NMR (500 MHz, CHCl_3_-d): δ = 32.88, 63.15, 111.07, 112.25, 119.11, 123.02, 126.53, 128.02, 128.25, 150.44, 162.13, 164.12, 171.02. MS: *m*/*z* = 400 (M^+^, 39%), 363 (100%), 321 (32%), 289 (88%), 254 (13%), 227 (10%), 215 (22%), 169 (23%), 153 (66%), 107 (18%).

#### 3.4.5. Synthesis of 3-(6-Fluoro-benzo[d]thiazol-2-yl)-2-(4-fluorophenyl)thiazolidin-4-one (**3**)

Yield: 21%, m.p. 192–193 °C, R_f_: 0.71 (petroleum ether/ethyl acetate 8:2). IR (Nujol), cm^−1^: 1705 (C=O), 1571, 1231, 1105. ^1^H-NMR (500 MHz, CHCl_3_-d): δ = 3.85 (d, *J* = 16.38 Hz, 1H, CH_2_), 4.11 (d, *J* = 16.63 Hz, 1H, CH_2_), 6.77 (d, *J* = 18.59 Hz, 1H, N-CH-S), 7.02 (t, *J* = 8.56 Hz, 1H, Ar-C5), 7.06–7.15 (m, 1H, Ar-C5), 7.27–7.36 (m, 4H, Ar-C17, C18, C20, C21), 7.48 (d, *J* = 8.02 Hz, 1H, Ar-C7). ^13^C-NMR (500 MHz, CHCl_3_-d): δ = 32.79, 63.09, 109.98, 113.94, 115.84, 120.83, 127.49, 127.55, 130.01, 133.32, 146.77, 156.13, 161.98, 163.52, 170.87 (1C, C=O). MS: *m*/*z* = 349 (M^+^, 7%), 288 (3%), 272 (25%), 245 (14%), 244 (100%), 240 (11%), 200 (7%), 163 (5%), 130 (6%).

#### 3.4.6. Synthesis of 3-(6-Fluoro-benzo[d]thiazol-2-yl)-2-(4-nitrophenyl)thiazolidin-4-one (**4**)

Yield: 55%, m.p. 204–205 °C, R_f_: 0.44 (petroleum ether/ethyl acetate 8:2). IR (Nujol), cm^−1^: 1706 (C=O), 1572, 1235. ^1^H-NMR (500 MHz, CHCl_3_-d): δ = 4.04 (d, *J* = 16.63 Hz, 1H, CH_2_), 4.29 (d, *J* = 16.63 Hz, 1H, CH_2_), 6.97 (s, 1H, N-CH-S), 7.22 (t, *J* = 8.43 Hz, 1H, Ar-C5), 7.62 (br s, 1H, Ar-C4), 7.68 (d, *J* = 7.34 Hz, 2H, Ar-C17, C21), 7.92 (br d, *J* = 8.80 Hz, 1H, Ar-C7), 8.17 (d, *J* = 8.31 Hz, 2H, Ar-C18, C20). ^13^C-NMR (500 MHz, CHCl_3_-d): δ = 32.77, 63.08, 109.95, 113.97, 117.94, 120.83, 123.32, 127.49(2C), 133.32, 145.78, 146.77, 156.12, 163.56, 170.96. MS: *m*/*z* = 376 (M^+^, 100%), 342 (25%), 272 (41%), 150 (38%), 115 (17%), 73 (11%).

#### 3.4.7. Synthesis of 3-(6-Fluoro-benzo[d]thiazol-2-yl)-2-(4-chlorophenyl)thiazolidin-4-one (**5**)

Yield: 74%, m.p. 175–176 °C, R_f_: 0.67 (petroleum ether/ethyl acetate 8:2). IR (Nujol), cm^−1^: 1708 (C=O), 1554, 1231, 721. ^1^H-NMR (500 MHz, CHCl_3_-d): δ = 3.86 (d, *J* = 16.38 Hz, 1H, CH_2_), 4.10 (br d, *J* = 16.63 Hz, 1H, CH_2_), 6.73 (s, 1H, N-CH-S), 7.06–7.19 (m, 1H, Ar-C5), 7.22–7.40 (m, 4H, Ar-C17, C18, C20, C21), 7.48 (br d, *J* = 7.83 Hz, 1H, Ar-C4), 7.56–7.79 (m, 1H, Ar-C7). ^13^C-NMR (500 MHz, CHCl_3_-d): δ = 32.79, 63.09, 109.98, 113.94, 117.94, 127.94, 130.32, 131.31, 132.35, 137.38, 146.77, 156.13, 163.52, 170.87. MS: *m*/*z* = 365 (M^+^, 100%), 343 (47%), 320 (7%), 291 (70%), 279 (34%), 259 (32%), 225 (28%).

#### 3.4.8. Synthesis of 3-(6-Fluoro-benzo[d]thiazol-2-yl)-2-(4-methoxyphenyl)thiazolidin-4-one (**6**)

Yield: 29%, m.p. 198–200 °C, R_f_: 0.63 (petroleum ether/ethyl acetate 8:2). IR (Nujol), cm^−1^: 1706 (C=O), 1572, 1230, 1213. ^1^H-NMR (500 MHz, CHCl_3_-d): δ = 3.76 (s, 3H, O-CH_3_), 3.91 (d, *J* = 16.38 Hz, 1H, CH_2_), 4.10 (d, *J* = 16.63 Hz, 1H, CH_2_), 6.73 (s, 1H, N-CH-S), 6.84 (d, *J* = 8.80 Hz, 2H, Ar-C18, C20), 7.12 (t, *J* = 8.56 Hz, 1H, Ar-C5), 7.23–7.35 (m, 1H, Ar-C4), 7.46–7.55 (m, 2H, Ar-C17, C21), 7.58–7.79 (m, 1H, Ar-C7). ^13^C-NMR (500 MHz, CHCl_3_-d): δ = 32.78, 58.01, 63.08, 109.98, 113.89, 114.13, 117.94, 129.49, 131.98, 132.35, 137.38, 146.73, 156.1, 158.01, 163.51, 171.03. MS: *m*/*z* = 361 (M^+^, 15%), 288 (9%), 272 (25%), 244 (100%), 243 (10%), 153 (13%), 135 (7%).

#### 3.4.9. Synthesis of 3-(6-Fluoro-benzo[d]thiazol-2-yl)-2-(4-hydroxyphenyl)thiazolidin-4-one (**7**)

Yield: 21%, m.p. 230–231 °C, R_f_: 0.37 (petroleum ether/ethyl acetate 8:2). IR (Nujol), cm^−1^: 3258 (-OH), 2361, 1706 (C=O), 1573, 1230. ^1^H-NMR (500 MHz, CHCl_3_-d): 4.04 (d, *J* = 16.63 Hz, 1H, CH_2_), 4.29 (d, *J* = 16.63 Hz, 1H, CH_2_), 5.35 (br s, 1H, -OH), 6.97 (s, N-CH-S), 7.22 (t, *J* = 8.43 Hz, 1H, Ar-C5), 7.62 (br s, 1H, Ar-C4), 7.68 (d, *J* = 7.34 Hz, 2H, Ar-C18, C20), 7.92 (br d, *J* = 8.80 Hz, 1H, Ar-C7), 8.16 (d, *J* = 8.31 Hz, 2H, Ar-C17, C21). ^13^C-NMR (500 MHz, CHCl_3_-d): δ = 32.81, 63.11, 109.94, 113.91, 115.12, 117.94, 130.00, 131.24, 131.53, 146.68, 156.09, 158.04, 163.52, 171.05. MS: *m*/*z* = 347 (M^+^, 42%), 309 (92%), 279 (40%), 263 (70%), 243 (72%).

#### 3.4.10. Synthesis of 3-(6-Chloro-benzo[d]thiazol-2-yl)-2-(4-fluorophenyl)thiazolidin-4-one (**8**)

Yield: 51%, m.p. 196–197 °C, R_f_: 0.76 (petroleum ether/ethyl acetate 8:2). IR (Nujol), cm^−1^: 2363, 1704 (C=O), 1568, 721. ^1^H-NMR (500 MHz, CHCl_3_-d): δ = 3.85 (d, *J* = 16.38 Hz, 1H, CH_2_), 4.11 (d, *J* = 16.63 Hz, 1H, CH_2_), 6.75 (s, 1H, N-CH-S), 7.10–7.18 (m, 2H, Ar-C18, C20), 7.27–7.36 (m, 2H, Ar-C17, C21), 7.56 (t, *J* = 8.56 Hz, 1H, Ar-C5), 7.61 (d, *J* = 8.02 Hz, 1H, Ar-C4), 8.05 (s, 1H, Ar-C7). ^13^C-NMR (500 MHz, CHCl_3_-d): δ = 32.86, 63.10, 115.20, 120.82, 122.73, 126.76, 127.73, 133.30, 136.08, 146.90, 156.05, 163.49, 171.11. MS: *m*/*z* = 364 (M^+^, 100%), 343 (10%), 299 (10%), 290 (100%), 279 (9%), 184 (11%), 115 (80%), 88 (9%), 73 (94%).

#### 3.4.11. Synthesis of 3-(6-Chloro-benzo[d]thiazol-2-yl)-2-(4-hydroxyphenyl)thiazolidin-4-one (**9**)

Yield: 24%, m.p. 236–237 °C, R_f_: 0.40 (petroleum ether/ethyl acetate 8:2). IR (Nujol), cm^−1^: 2359 (-OH), 2361, 1701 (C=O), 1573. ^1^H-NMR (500 MHz, CHCl_3_-d): δ = 3.86 (d, *J* = 16.38 Hz, 1H, CH_2_), 4.10 (d, *J* = 16.63 Hz, 1H, CH_2_), 5.35 (br s, 1H, -OH), 6.73 (s, 1H, N-CH-S), 7.26–7.43 (m, 5H, Ar-C5, C17, C18, C20, C21), 7.76 (br d, *J* = 8.80 Hz, 1H, Ar-C4), 8.13 (s, 1H, Ar-C7). ^13^C-NMR (500 MHz, CHCl_3_-d): δ = 33.12, 63.71, 115.75, 118.23, 121.55, 125.14, 129.02, 130.01, 131.52, 132.87, 151.68, 156.71, 163.77, 171.12. MS: *m*/*z* = 363 (M^+^, 100%), 288 (88%), 272 (9%), 225 (33%), 184 (48%), 146 (7%), 136 (21%), 115 (18%), 73 (17%).

#### 3.4.12. Synthesis of 3-(4-Chloro-benzo[d]thiazol-2-yl)-2-(4-fluorophenyl)thiazolidin-4-one (**10**)

Yield: 33%, m.p. 215–216 °C, R_f_: 0.74 (petroleum ether/ethyl acetate 8:2). IR (Nujol), cm^−1^: 2361, 1702 (C=O), 1573, 1101. ^1^H-NMR (500 MHz, CHCl_3_-d): δ = 3.93 (d, *J* = 16.63 Hz, 1H, CH_2_), 4.21 (d, *J* = 16.63 Hz, 1H, CH_2_), 6.81 (s, 1H, N-CH-S), 7.02 (br t, *J* = 8.56 Hz, 2H, Ar-C18, C20), 7.15–7.24 (m, 1H, Ar-C6), 7.41 (d, *J* = 7.34 Hz, 1H, Ar-C5), 7.50 (br dd, *J* = 8.31, 5.38 Hz, 2H, Ar-C17, C21), 7.68 (d, *J* = 7.83 Hz, 1H, Ar-C7). ^13^C-NMR (500 MHz, CHCl_3_-d): δ = 32.81, 63.87, 115.02, 119.33, 120.77, 121.13, 125.76, 130.71, 132.45, 134.38, 149.11, 161.33, 164.02, 171.05. MS: *m*/*z* = 364 (M^+^, 72%), 343 (35%), 232 (31%), 290 (100%), 292 (79%), 279 (33%), 242 (64%), 225 (65%), 184 (77%), 174 (44%).

#### 3.4.13. Synthesis of 3-(4-Chloro-benzo[d]thiazol-2-yl)-2-(4-nitrophenyl)thiazolidin-4-one (**11**)

Yield: 41%, m.p. 203–204 °C, R_f_: 0.45 (petroleum ether/ethyl acetate 8:2). IR (Nujol), cm^−1^: 2361, 1701 (C=O), 1573. ^1^H-NMR (500 MHz, CHCl_3_-d): δ = 3.93 (d, *J* = 16.63 Hz, 1H, CH_2_), 4.21 (d, *J* = 16.63 Hz, 1H, CH_2_), 6.85 (s, 1H, N-CH-S), 7.21–7.30 (d, *J* = 7.34 Hz, 2H, Ar-C17, C21), 7.40 (br d, *J* = 8.80 Hz, 1H, Ar-C6), 7.80 (br d, *J* = 8.80 Hz, 1H, Ar-C5), 8.20 (d, *J* = 8.31 Hz, 3H, Ar-C7, C18, C20). ^13^C-NMR (500 MHz, CHCl_3_-d): δ = 32.86, 63.35, 119.32, 121.14, 121.58, 123.69, 125.81, 129.66, 132.11, 145.38, 146.37, 149.72, 164.01, 171.02. MS: *m*/*z* = 373 (M^+^, 33%), 349(14%), 343 (46%), 332 (40%), 304 (29%), 292 (92%), 290 (100%), 279 (18%), 259 (7%).

#### 3.4.14. Synthesis of 3-(4-Chloro-benzo[d]thiazol-2-yl)-2-(4-chlorophenyl)thiazolidin-4-one (**12**)

Yield: 56%, m.p. 184–185 °C, R_f_: 0.73 (petroleum ether/ethyl acetate 8:2). IR (Nujol), cm^−1^: 2361, 1704 (C=O), 1568, 721. ^1^H-NMR (500 MHz, CHCl_3_-d): δ = 3.94 (d, *J* = 16.63 Hz, 1H, CH_2_), 4.21 (d, *J* = 16.63 Hz, 1H, CH_2_), 6.79 (s, 1H, N-CH-S), 7.21–7.31 (m, 3H, Ar-C6, C17, C21), 7.39–7.43 (m, 3H, Ar-C5, C18, C20), 7.68 (d, *J* = 7.83 Hz, 1H, Ar-C7). ^13^C-NMR (500 MHz, CHCl_3_-d): δ = 32.83, 63.12, 119.89, 121.23, 121.59, 125.83, 128.77, 130.77, 132.31, 132.59, 145.38, 137.36, 149.71, 163.95, 171.03. MS: *m*/*z* = 383 (M^+^, 13%), 381 (43%), 379 (61%), 346 (11%), 341 (11%), 337 (18%), 269 (46%), 227 (32%).

#### 3.4.15. Synthesis of 3-(4-Chloro-benzo[d]thiazol-2-yl)-2-(4-methoxyphenyl)thiazolidin-4-one (**13**)

Yield: 67%, m.p. 223–224 °C, R_f_: 0.54 (petroleum ether/ethyl acetate 8:2). IR (Nujol), cm^−1^: 2361, 1700 (C=O), 1571, 1232. ^1^H-NMR (500 MHz, CHCl_3_-d): δ = 3.75 (s, 3H, O-CH_3_), 3.92 (d, *J* = 16.38 Hz, 1H, CH_2_), 4.20 (d, *J* = 16.63 Hz, 1H, CH_2_), 6.81 (s, 1H, N-CH-S), 6.84(d, *J* = 8.80 Hz, 2H, Ar-C18, C20), 7.19 (t, *J* = 8.56 Hz, 1H, Ar-C6), 7.25 (s, 1H, Ar-C5), 7.41–7.45 (m, 2H, Ar-C17, C21), 7.68 (d, *J* = 7.83 Hz, 1H, Ar-C7). ^13^C-NMR (500 MHz, CHCl_3_-d): δ = 32.85, 55.81, 63.32, 114.21, 119.31, 121.18, 121.57, 125.86, 129.79, 131.28, 132.38, 149.49, 159.00, 163.52, 171.04. MS: *m*/*z* = 377 (M^+^, 100%), 362 (15%), 353 (31%), 326 (27%), 290 (40%), 288 (82%), 136 (36%), 115 (17%) 73 (14%).

#### 3.4.16. Synthesis of 3-(4-Chloro-benzo[d]thiazol-2-yl)-2-(4-hydroxyphenyl)thiazolidin-4-one (**14**)

Yield: 17%, m.p. 258–259 °C, R_f_: 0.39 (petroleum ether/ethyl acetate 8:2). IR (Nujol), cm^−1^: 3205 (-OH), 2361, 1701 (C=O), 1569. ^1^H-NMR (500 MHz, CHCl_3_-d): δ = 3.93 (d, *J* = 16.63 Hz, 1H, CH_2_), 4.21 (d, *J* = 16.63 Hz, 1H, CH_2_), 5.32 (br s, 1H, -OH), 6.81 (s, 1H, N-CH-S), 7.02 (br t, *J* = 8.56 Hz, 2H, Ar-C18, C20), 7.15–7.24 (m, 1H, Ar-C6), 7.41 (d, *J* = 7.34 Hz, 1H, Ar-C5), 7.50 (br dd, *J* = 8.31, 5.38 Hz, 2H, Ar-C17, C21), 7.68 (d, *J* = 7.83 Hz, 1H, Ar-C7). ^13^C-NMR (500 MHz, CHCl_3_-d): δ = 33.02, 63.31, 115.18, 119.23, 121.55, 121.89, 125.15, 130.03, 131.52, 132.17, 149.68, 156.73, 163.79, 171.08. MS: *m*/*z* = 363 (M^+^, 75%), 346 (28%), 310 (22%), 290 (21%), 272 (100%), 184 (19%), 136 (22%), 115 (51%), 88 (27%), 73 (15%).

#### 3.4.17. Synthesis of 3-(4-Methoxy-benzo[d]thiazol-2-yl)-2-(4-flurophenyl)thiazolidin-4-one (**15**)

Yield: 51%, m.p. 251–252 °C, R_f_: 0.73 (petroleum ether/ethyl acetate 8:2). IR (Nujol), cm^−1^: 2361, 1968 (C=O), 1587, 1272, 1100. ^1^H-NMR (500 MHz, DMSO-d_6_): δ = 3.68–3.86 (m, 3H, O-CH_3_), 3.91–4.09 (m, 1H, CH_2_), 4.25 (br d, *J* = 9.78 Hz, 1H, CH_2_), 6.88 (s, 1H, N-CH-S), 7.06 (br d, *J* = 10.27 Hz, 1H, Ar-C5), 7.26 (br dd, *J* = 10.03, 8.07 Hz, 1H, Ar-C6), 7.55 (br d, *J* = 10.27 Hz, 1H, Ar-C7), 7.61–7.76 (m, 2H, Ar-C17, C21), 8.08–8.26 (m, 2H, Ar-C18, C20). ^13^C-NMR (500 MHz, DMSO-d_6_): δ = 32.86, 55.82, 63.18, 108.26, 114.11, 115.43, 121.18, 130.33, 131.99, 135.59, 142.31, 150.23, 161.51, 163.91, 171.08. MS: *m*/*z* = 361 (M^+^, 100%), 343 (42%), 321 (36%), 290 (72%), 288 (31%), 279 (39%), 225 (24%).

#### 3.4.18. Synthesis of 3-(4-Methoxy-benzo[d]thiazol-2-yl)-2-(4-nitrophenyl)thiazolidin-4-one (**16**)

Yield: 63%, m.p. 298–299 °C, R_f_: 0.48 (petroleum ether/ethyl acetate 8:2). IR (Nujol), cm^−1^: 2361, 1705 (C=O), 1585, 1273. ^1^H-NMR (500 MHz, DMSO-d_6_): δ = 3.77 (s, 3H, O-CH_3_), 4.03 (d, *J* = 17.12 Hz, 1H, CH_2_), 4.20 (d, *J* = 17.12 Hz, 1H, CH_2_), 6.93 (s, 1H, N-CH-S), 7.05 (s, 1H, Ar-C5), 7.26 (s, 1H, Ar-C6), 7.56 (d, *J* = 7.83 Hz, 2H, Ar-C17, C21), 7.67 (d, *J* = 8.80 Hz, 1H, Ar-C7), 8.17 (d, *J* = 8.32 Hz, 2H, Ar-C18, C20). ^13^C-NMR (500 MHz, DMSO-d_6_): δ = 32.85, 55.85, 63.21, 108.25, 114.12, 121.18, 123.84, 129.66, 131.97, 142.31, 145.26, 146.32, 150.21, 163.95, 171.02. MS: *m*/*z* = 388 (M^+^, 100%), 288 (73%), 272 (3%), 225 (42%), 184 (76%), 136 (42%), 115 (31%), 73 (28%).

#### 3.4.19. Synthesis of 3-(4-Methoxy-benzo[d]thiazol-2-yl)-2-(4-chlorophenyl)thiazolidin-4-one (**17**)

Yield: 43%, m.p. 218–219 °C, R_f_: 0.53 (petroleum ether/ethyl acetate 8:2). IR (Nujol), cm^−1^: 2361, 1708 (C=O), 1589, 1276, 1154. ^1^H-NMR (500 MHz, DMSO-d_6_): δ = 3.80 (s, 3H, O-CH_3_), 3.99 (br d, *J* = 17.12 Hz, 1H, CH_2_), 4.23 (br d, *J* = 16.63 Hz, 1H, CH_2_), 6.93 (s, 1H, N-CH-S), 6.91–6.97 (m, 1H, Ar-C5), 7.26 (br t, *J* = 8.07 Hz, 1H, Ar-C4), 7.39 (br d, *J* = 9.29 Hz, 4H, Ar-C17, C18, C20, C21), 7.54 (br d, *J* = 7.83 Hz, 1H, Ar-C7). ^13^C-NMR (500 MHz, DMSO-*d*_6_): δ = 32.82, 55.88, 63.27, 108.25, 114.11, 121.32, 128.73, 130.12, 131.93, 132.75, 137.36, 142.32, 150.22, 164.03, 171.00. MS: *m*/*z* = 377 (M^+^, 41%), 272 (23%), 244 (100%), 242 (63%), 165 (17%), 136 (23%).

#### 3.4.20. Synthesis of 3-(4-Methoxy-benzo[d]thiazol-2-yl)-2-(4-methoxyphenyl)thiazolidin-4-one (**18**)

Yield: 52%, m.p. 196–197 °C, R_f_: 0.63 (petroleum ether/ethyl acetate 8:2). IR (Nujol), cm^−1^: 2361, 1701 (C=O), 1556, 1273. ^1^H-NMR (500 MHz, CHCl_3_-d): δ = 33.76 (s, 3H, O-CH_3benz_), 3.94 (d, *J* = 16.63 Hz, 1H, CH_2_), 3.95 (s, 3H, O-CH_3_), 4.13 (d, *J* = 16.63 Hz, 1H, CH_2_), 6.81–6.85 (m, 2H, Ar-C18, C20), 7.93 (s, 1H, N-CH-S), 7.25 (br d, *J* = 9.29 Hz, 3H, Ar-C5, C6, C7), 7.39 (br d, *J* = 7.83 Hz, 2H, Ar-C17, C21). ^13^C-NMR (500 MHz, CHCl_3_-d): δ = 32.86, 55.83, 63.35, 108.25, 114.11, 114.76, 121.82, 129.75, 131.55, 131.96, 142.34, 150.23, 160.82, 164.06, 171.11. MS: *m*/*z* = 373 (M^+^, 76%), 272 (33%), 243 (56%), 244 (100%), 242 (8%), 165 (5%), 164 (7%), 135 (11%).

#### 3.4.21. Synthesis of 3-(4-Methoxy-benzo[d]thiazol-2-yl)-2-(4-hydroxyphenyl)thiazolidin-4-one (**19**)

Yield: 29%, m.p. 261–262 °C, R_f_: 0.72 (petroleum ether/ethyl acetate 8:2). IR (Nujol), cm^−1^: 3205 (-OH), 2361, 1707 (C=O), 1582, 1271. ^1^H-NMR (500 MHz, DMSO-d_6_): δ = 3.77 (s, 3H, O-CH_3_), 4.03 (d, *J* = 17.12 Hz, 1H, CH_2_), 4.20 (d, *J* = 17.12 Hz, 1H, CH_2_), 5.32 (br s, 1H, -OH), 6.93 (d, *J* = 7.83 Hz, 2H, Ar-C18, C20), 7.05 (s, 1H, N-CH-S), 7.26 (br t, *J* = 8.56 Hz, 1H, Ar-C5), 7.56 (d, *J* = 7.83 Hz, 1H, Ar-C6), 7.67 (d, *J* = 8.80 Hz, 1H, Ar-C7), 8.17 (d, *J* = 8.32 Hz, 2H, Ar-C17, C21). ^13^C-NMR (500 MHz, CHCl_3_-d): δ = 32.88, 55.82, 63.33, 108.21, 114.13, 115.81, 121.73, 130.13, 131.52, 131.96, 142.31, 150.22, 156.95, 164.13, 171.04. MS: *m*/*z* = 359 (M^+^, 82%), 344 (30%), 328 (64%), 189 (27%), 244 (100%), 165 (54%), 135 (21%).

#### 3.4.22. Synthesis of 3-(6-Methoxy-benzo[d]thiazol-2-yl)-2-(4-fluorophenyl)thiazolidin-4-one (**20**)

Yield: 52%, m.p. 242–243 °C, R_f_: 0.62 (petroleum ether/ethyl acetate 8:2). IR (Nujol), cm^−1^: 2361, 1701 (C=O), 1531, 1225. ^1^H-NMR (500 MHz, DMSO-d_6_): δ = 3.76 (s, 3H, O-CH_3_), 3.99 (br d, *J* = 16.63 Hz, 1H, CH_2_), 4.26 (br d, *J* = 16.63 Hz, 1H, CH_2_), 6.84 (s, 1H, N-CH-S), 6.97 (dd, *J* = 8.80, 2.45 Hz, 1H, Ar-C5), 7.13 (br t, *J* = 8.80 Hz, 2H, Ar-C18, C20), 7.44 (br dd, *J* = 8.31, 5.38 Hz, 2H, Ar-C17, C21), 7.49–7.71 (m, 2H, Ar-C4, C7). ^13^C-NMR (500 MHz, DMSO-d_6_): δ = 32.96, 55.82, 63.33, 108.21, 114.13, 115.81, 121.73, 130.13, 131.52, 131.96, 142.31, 150.22, 156.95, 164.13, 171.04. MS: *m*/*z* = 361 (M^+^, 14%), 288 (8%), 272 (25%), 260 (5%), 258 (8%), 244 (100%), 243 (8%), 153 (13%), 165 (4%), 135 (7%), 109 (8%).

#### 3.4.23. Synthesis of 3-(6-Methoxy-benzo[d]thiazol-2-yl)-2-(4-nitrophenyl)thiazolidin-4-one (**21**)

Yield: 41%, m.p. 220–221 °C, R_f_: 0.49 (petroleum ether/ethyl acetate 8:2). IR (Nujol), cm^−1^: 2361, 1701 (C=O), 1532, 1226. ^1^H-NMR (500 MHz, DMSO-d_6_): δ = 3.76 (s, 3H, O-CH_3_), 4.03 (br d, *J* = 16.63 Hz, 1H, CH_2_), 4.27 (br d, *J* = 16.63Hz, 1H, CH_2_), 6.89–7.06 (m, 2H, Ar-C5, N-CH-S), 7.51 (br d, *J* = 9.29 Hz, 1H, Ar-C5), 7.60 (br s, 1H, Ar-C7), 7.67 (br d, *J* = 7.34 Hz, 2H, Ar-C17, C21), 8.17 (br d, *J* = 8.31 Hz, 2H, Ar-C18, C20). ^13^C-NMR (500 MHz, DMSO-d_6_): δ = 32.87, 55.86, 63.32, 104.12, 114.33, 117.25, 124.38, 125.96, 131.95, 145.13, 145.51, 146.79, 156.81, 164.03, 170.92. MS: *m*/*z* = 388 (M^+^, 62%), 288 (100%), 225 (29%), 184 (61%), 136 (27%), 115 (23%), 107 (27%), 73 (18%).

#### 3.4.24. Synthesis of 3-(6-Methoxy-benzo[d]thiazol-2-yl)-2-(4-chlorophenyl)thiazolidin-4-one (**22**)

Yield: 52%, m.p. 179–180 °C, R_f_: 0.53 (petroleum ether/ethyl acetate 8:2). IR (Nujol), cm^−1^: 2361, 1706 (C=O), 1536, 1219, 1108. ^1^H-NMR (500 MHz, DMSO-*d*_6_): δ = 3.77 (s, 3H, O-CH_3_), 3.99 (d, *J* = 17.12 Hz, 1H, CH_2_), 4.26 (d, *J* = 16.63 Hz, 1H, CH_2_), 6.84 (s, 1H, N-CH-S), 6.97 (dd, *J* = 8.80, 2.45 Hz, 1H, Ar-C5), 7.32–7.47 (m, 4H, Ar-C17, C18, C20, C21), 7.47–7.65 (m, 2H, Ar-C4, C7). ^13^C-NMR (500 MHz, DMSO-d_6_): δ = 32.76, 55.85, 63.31, 104.99, 114.61, 118.23, 127.81(2C), 129.16, 131.93, 132.15, 137.33, 145.52, 156.74, 164.03, 170.85. MS: *m*/*z* = 377 (M^+^, 100%), 288 (82%), 272 (33%), 225 (21%), 136 (35%), 115 (20%), 73 (18%).

#### 3.4.25. Synthesis of 3-(6-Methoxy-benzo[d]thiazol-2-yl)-2-(4-methoxyphenyl)thiazolidin-4-one (**23**)

Yield: 33%, m.p. 225–226 °C, R_f_: 0.62 (petroleum ether/ethyl acetate 8:2). IR (Nujol), cm^−1^: 2361, 1700 (C=O), 1540, 1225. ^1^H-NMR (500 MHz, CHCl_3_-d): δ = 3.76 (s, 3H, O-CH_3benz_), 3.94 (d, *J* = 16.63 Hz, 1H, CH_2_), 3.98 (s, 3H, O-CH_3_), 4.13 (d, *J* = 16.63 Hz, 1H, CH_2_), 6.84 (s, 1H, N-CH-S), 6.93 (br d, *J* = 8.31 Hz, 2H, Ar-C18, C20), 7.51 (br d, *J* = 9.29 Hz, 1H, Ar-C5), 7.25 (br d, *J* = 9.29 Hz, 2H, Ar-C4, C7, C21), 7.39 (br d, *J* = 7.83 Hz, 1H, Ar-C17). ^13^C-NMR (500 MHz, CHCl_3_-d): δ = 32.81, 55.84, 63.33, 104.92, 114.81, 115.01, 118.22, 127.79, 131.91, 132.28, 145.55, 156.72, 159.85, 163.92, 170.93. MS: *m*/*z* = 373 (M^+^, 78%), 299 (10%), 344 (10%), 272 (33%), 244 (100%), 242 (7%), 165 (5%), 164 (7%), 150 (9%), 135 (3%).

#### 3.4.26. Synthesis of 3-(6-Ethoxy-benzo[d]thiazol-2-yl)-2-(4-fluorophenyl)thiazolidin-4-one (**24**)

Yield: 63%, m.p. 144–145 °C, R_f_: 0.44 (petroleum ether/ethyl acetate 8:2). IR (Nujol), cm^−1^: 2316, 1701 (C=O), 1531, 1225. ^1^H-NMR (500 MHz, CHCl_3_-d): δ = 1.25 (t, *J* = 6.85 Hz, 3H, -OCH_2_CH_3_), 3.82–3.85 (m, 3H, CH_2,_-OCH_2_CH_3_), 4.10 (d, *J* = 16.63 Hz, 1H, CH_2_), 6.79 (s, 1H, N-CH-S), 6.97 (dd, *J* = 8.80, 2.45 Hz, 2H, Ar-C18, C20), 7.25 (s, 1H, Ar-C5), 7.30–7.33 (m, 2H, Ar-C17, C21), 7.59 (d, *J* = 8.80 Hz, 2H, Ar-C4, C7). ^13^C-NMR (500 MHz, CHCl_3_-d): δ = 14.13, 32.35, 55.89, 63.12, 104.52, 115.13, 115.52, 115.61, 130.02, 131.51, 134.03, 144.00, 153.62, 161.13, 164.05, 171.21. MS: *m*/*z* = 375 (M^+^, 100%), 288 (28%), 272 (36%), 225 (18%), 197 (19%), 136 (20%), 115 (16%), 73 (16%).

#### 3.4.27. Synthesis of 3-(6-Ethoxy-benzo[d]thiazol-2-yl)-2-(4-nitrophenyl)thiazolidin-4-one (**25**)

Yield: 72%, m.p. 155–156 °C, R_f_: 0.58 (petroleum ether/ethyl acetate 8:2). IR (Nujol), cm^−1^: 2316, 1701 (C=O), 1527, 1225. ^1^H-NMR (500 MHz, CHCl_3_-d): δ = 1.42 (t, *J* = 6.85 Hz, 3H, -OCH_2_CH_3_), 3.89 (d, *J* = 16.63 Hz, 1H, CH_2_), 4.10 (d, *J* = 16.63 Hz, 1H, CH_2_), 4.25–4.32 (q, *J* = 6.68 Hz, 2H, -OCH_2_CH_3_), 6.80 (s, 1H, N-CH-S), 6.97 (dd, *J* = 8.80, 2.45 Hz, 1H, Ar-C5), 7.19–7.33 (m, 1H, Ar-C7), 7.42–7.59 (m, 3H, Ar-C4, C17, C21), 8.19 (d, *J* = 8.80 Hz, 2H, Ar-C18, C20). ^13^C-NMR (500 MHz, CHCl_3_-d): δ = 14.12, 32.31, 55.97, 63.17, 104.52, 115.25, 117.31, 122.15, 129.75, 131.21, 142.02, 143.85, 145.06, 153.72, 164.02, 170.75. MS: *m*/*z* = 402 (M^+^, 100%), 382 (49%), 373 (42%), 357 (21%), 348 (18%), 115 (12%), 73 (7%).

#### 3.4.28. Synthesis of 3-(6-Ethoxy-benzo[d]thiazol-2-yl)-2-(4-chlorophenyl)thiazolidin-4-one (**26**)

Yield: 82%, m.p. 180–181°C, R_f_: 0.66 (petroleum ether/ethyl acetate 8:2). IR (Nujol), cm^−1^: 2361, 1696 (C=O), 1546, 1227, 698. ^1^H-NMR (500 MHz, CHCl_3_-d): δ = 1.43 (t, *J* = 6.85 Hz, 3H, -OCH_2_CH_3_), 3.83 (d, *J* = 16.63 Hz, 1H, CH_2_), 4.04–4.10 (m, 3H, CH_2,_ -OCH_2_CH_3_), 6.74 (s, 1H, N-CH-S), 6.98 (dd, *J* = 8.80, 2.45 Hz, 1H, Ar-C5), 7.25–7.30 (m, 5H, Ar-c4, C17, C18, C20, C21), 7.59 (d, *J* = 8.80 Hz, 1H, Ar-C7). ^13^C-NMR (500 MHz, CHCl_3_-d): δ = 14.12, 32.31, 55.92, 63.17, 104.83, 114.77, 117.81, 128.72, 130.05, 131.24, 132.74, 137.52, 144.86, 145.06, 153.59, 164.07, 170.69. MS: *m*/*z* = 391 (M^+^, 100%), 363 (68%), 272 (88%), 244 (67%), 215 (26%), 153 (79%).

#### 3.4.29. Synthesis of 3-(6-Ethoxy-benzo[d]thiazol-2-yl)-2-(4-methoxyphenyl)thiazolidin-4-one (**27**)

Yield: 55%, m.p. 210–211°C, R_f_: 0.63 (petroleum ether/ethyl acetate 8:2). IR (Nujol), cm^−1^: 2361, 1700 (C=O), 1531, 1227. ^1^H-NMR (500 MHz, CHCl_3_-d): δ = 1.42 (t, *J* = 6.85 Hz, 3H, -OCH_2_CH_3_), 3.89 (d, *J* = 16.63 Hz, 1H, CH_2_), 4.03–4.10 (m, 4H, CH_2_, -OCH_3_), 4.25–4.32 (q, *J* = 6.68 Hz, 2H, -OCH_2_CH_3_), 6.80 (s, 1H, N-CH-S), 6.97 (dd, *J* = 8.80, 2.45 Hz, 2H, Ar-C18, C20), 7.19–7.33 (m, 1H, Ar-C5), 7.42–7.59 (m, 2H, Ar-C4, C7), 8.19 (d, *J* = 8.80 Hz, 2H, Ar-C17, C21). ^13^C-NMR (500 MHz, CHCl_3_-d): δ = 14.15, 32.36, 55.81, 55.96, 63.17, 105.26, 114.21, 114.75, 117.82, 129.85, 131.58, 144.82, 153.56, 159.35, 164.15, 170.57. MS: *m*/*z* = 387 (M^+^, 21%), 344 (16%), 313 (15%), 299 (8%), 272 (37%), 244 (100%), 242 (15%), 165 (35%), 164 (9%), 150 (23%), 135 (8%).

#### 3.4.30. Synthesis of 3-(6-Ethoxy-benzo[d]thiazol-2-yl)-2-(4-hydroxyphenyl)thiazolidin-4-one (**28**)

Yield: 81%, m.p. 191–192°C, R_f_: 0.37 (petroleum ether/ethyl acetate 8:2). IR (Nujol), cm^−1^: 3200 (-OH), 2361, 1706 (C=O), 1530, 1225. ^1^H-NMR (500 MHz, DMSO-d_6_): δ = 1.33 (br t, *J* = 6.60 Hz, 3 H), 3.37–3.50 (m, 4 H), 3.51–3.68 (m, 1 H), 4.04 (q, *J* = 6.68 Hz, 2 H), 5.24 (s, 1 H), 6.71 (m, *J* = 7.83 Hz, 2 H), 6.99 (br d, *J* = 8.80 Hz, 1 H), 7.20 (m, *J* = 7.83 Hz, 2 H), 7.52 (br s, 1 H), 7.60 (br d, *J* = 8.31 Hz, 1 H), 9.55 (br s, 1 H). ^13^C-NMR (500 MHz, DMSO-*d*_6_): δ = 14.11, 32.31, 55.93, 63.19, 104.97, 114.67, 115.32, 117.82, 130.05, 130.87, 132.56, 144.81, 154.59, 156.73, 164.07, 170.63. MS: *m*/*z* = 373 (M^+^, 9%), 288 (17%), 272 (29%), 244 (100%), 243 (8%), 197 (11%), 109 (4%).

#### 3.4.31. Synthesis of 3-(6-Trifluoromethoxy-benzo[d]thiazol-2-yl)-2-(2,6-dichlorophenyl) thiazolidin-4-one (**29**)

Yield: 73%, m.p. 164–165°C, R_f_: 0.53 (petroleum ether/ethyl acetate 8:2). IR (Nujol), cm^−1^: 2361, 1700 (C=O), 1532, 1209, 1134, 721. ^1^H-NMR (500 MHz, CHCl_3_-d): δ = 3.99 (d, *J* = 16.63 Hz, 1H, CH_2_), 4.26 (d, *J* = 16.63 Hz, 1H, CH_2_), 6.84 (s, 1H, N-CH-S), 6.97 (dd, *J* = 8.80, 2.45 Hz, 1H, Ar-C5), 7.42–7.46 (m, 3H, Ar-C18, C19, C20), 7.49–7.71 (m, 2H, Ar-C4, C7).^13^C-NMR (500 MHz, CHCl_3_-d): δ = 32.86, 63.33, 104.97, 114.22, 118.71, 126.82, 128.53, 129.27, 130.05, 135.11, 139.11, 145.91, 156.74, 164.13, 171.17. MS: *m*/*z* = 446 (M^+^, 47%), 340 (21%), 336 (16%), 321 (44%), 293 (100%), 292 (11%), 204 (46%), 165 (21%).

#### 3.4.32. Synthesis of 3-(6-Trifluoromethoxy-benzo[d]thiazol-2-yl)-2-(2,3-dichlorophenyl) thiazolidin-4-one (**30**)

Yield: 84%, m.p. 142–143 °C, R_f_: 0.81 (petroleum ether/ethyl acetate 8:2). IR (Nujol), cm^−1^: 2361, 1700 (C=O), 1532, 1202, 1131, 721. ^1^H-NMR (500 MHz, CHCl_3_-d): δ = 3.94 (d, *J* = 16.63 Hz, 1H, CH_2_), 4.11 (d, *J* = 16.63 Hz, 1H, CH_2_), 6.83 (s, 1H, N-CH-S), 6.91–7.01 (m, 2H, Ar-C5, C17), 7.10–7.18 (m, 1H, Ar-C18), 7.49–7.53 (m, 2H, Ar-C4, C7), 7.60 (dd, *J* = 8.80, 2.45 Hz, 1H, Ar-C19). ^13^C-NMR (500 MHz, CHCl_3_-d): δ = 32.85, 63.34, 103.85, 104.92, 114.66, 118.97, 127.95, 128.05, 128.77, 129.23, 129.56, 131.81, 132.05, 145.52, 156.73, 164.13, 170.93. MS: *m*/*z* = 466 (M^+^, 3%), 355 (6%), 272 (18%), 244 (36%), 228 (15%), 226 (22%), 165 (100%).

#### 3.4.33. Synthesis of 3-(6-Trifluoromethoxy-benzo[d]thiazol-2-yl)-2-(4-nitrophenyl)thiazolidin-4-one (**31**)

Yield: 33%, m.p. 158–159 °C, R_f_: 0.62 (petroleum ether/ethyl acetate 8:2). IR (Nujol), cm^−1^: 1706(C=O), 1539, 1209, 1135. ^1^H-NMR (500 MHz, CHCl_3_-d): δ = 3.85 (d, *J* = 16.63 Hz, 1H, CH_2_), 4.14 (d, *J* = 16.63 Hz, 1H, CH_2_), 6.76 (s, 1H, N-CH-S), 7.02 (d, *J* = 7.83 Hz, 1H, Ar-C5), 7.41–7.45 (m, 4H, Ar-C4, C7, C17, C21), 8.05 (d, *J* = 7.83 Hz, 2H, Ar-C18, C20). ^13^C-NMR (500 MHz, CHCl_3_-d): δ = 32.76, 63.09, 104.92, 114.02, 118.27 123.88, 129.12, 129.93, 131.95, 145.62, 145.94, 149.79, 156.71, 164.19, 170.92. MS: *m*/*z* = 442 (M^+^, 56%), 395 (51%), 386 (19%), 272 (100%), 244 (22%), 165 (7%).

#### 3.4.34. Synthesis of 3-(6-Trifluoromethoxy-benzo[d]thiazol-2-yl)-2-(4-chlorophenyl)thiazolidin-4- one (**32**)

Yield: 35%, m.p. 243–244 °C, R_f_: 0.77 (petroleum ether/ethyl acetate 8:2). IR (Nujol), cm^−1^: 1698 (C=O), 1528, 1209, 1198, 1131, 721. ^1^H-NMR (500 MHz, CHCl_3_-d): δ = 3.85 (d, *J* = 16.63 Hz, 1H, CH_2_), 4.10 (d, *J* = 16.63 Hz, 1H, CH_2_), 6.74 (s, 1H, N-CH-S), 7.24–7.31(m, 5H, Ar-C5, C17, C18, C20, C21), 7.66–7.71 (m, 2H, Ar-C4, C7). ^13^C-NMR (500 MHz, CHCl_3_-d): δ = 32.75, 63.05, 104.93, 114.01, 120.11, 128.27, 129.65, 129.81, 131.74, 132.92, 137.33, 145.95, 156.63, 161.62, 164.27, 170.94. MS: *m*/*z* = 431 (M^+^, 46%), 395 (27%), 378 (34%), 377 (100%), 373 (18%), 363 (55%).

### 3.5. Evaluation of RT Inhibitory Action

For the evaluation of the HIV-1 Reverse Transcriptase activity, the colorimetric photometric immunoassay kit provided by Roche was used. A two hours incubation at 37 °C was performed, as previously described in our paper [44] following a pre-incubation step in which reverse transcriptase was incubated in the presence of the potential inhibitor for 45 min at room temperature before the addition of the substrate. The total-activity was measured in the absence of inhibitors (compounds) with the addition of an equal volume of the solvent of the compounds to the reaction mixture. For the determination of enzyme activity, incubation in the absence of enzyme was used as blank. Different inhibitor concentrations were added for the calculation of IC_50_ values.

### 3.6. Toxicity

Cell culture and cytotoxicity assessment.

The normal MRC-5 cell line is stored and used in our laboratory in a routine manner. MRC-5 cells were grown in culture (37 °C, humidified atmosphere containing 5% *v*/*v* CO_2_) in DMEM medium supplemented with 10% *v*/*v* FBS, 100 μg/mL penicillin and 100 μg/mL streptomycin. The compounds under evaluation were dissolved in DMSO and stored in 4 °C (DMSO concentration ≤ 0.2% *v*/*v*). The cytotoxicity of the **1**, **2**, **3**, **8**, **9**, **10**, **14** compounds were evaluated in MRC-5 cell cultures. In particular cells were seeded in a 24- well plate at an initial concentration of 1 × 10^5^ cells/mL and allowed to stand overnight before the addition of the compounds at high concentration of 10^−5^ M. Note that DMSO concentration in culture was 0.2% *v*/*v*, where this concentration exhibits no cytotoxicity at all [50]. To estimate the cytotoxicity of each compound, cells were allowed to grow for additional 48h before being harvested by trypsinization and counted with the aid of the optical microscope (Neubauer counting chamber). Cell growth in each treated culture is expressed as a percentage compared to that seen for the untreated control cells. Moreover, cell death was also determined using the Trypan-blue method, as previously described [51,52]. Statistical t-test analysis was performed via the use of GraphPad Prism 6.0 program.

## 4. Conclusions

Thirty two compounds out of a hundred thirty two compounds designed were selected based on PASS prediction results and molecular docking for the synthesis and evaluation of HIV-1 RT inhibitory activity. Twenty-four out of the thirty-two tested compounds exhibited inhibitory action equal or lower than 4 μΜ. Seven of them showed better activity than nevirapine under experimental condition, while three of the compounds exhibited IC_50_ values lower than 5 nM. Two compounds **9** and **10** exhibited very good inhibitory activity with IC50 1 nM.

According to docking results, most of the compounds are placed within the NNRTIs binding pocket between the place equipped by the classic butterfly shaped compound, nevirapine and the binding site of etravirine or within the etravirine binding pocket.

It is interesting to mention that, the most active compounds structurally belong to three different groups and adopt three different orientations.

In general, according to docking studies and in vitro results, it seems that the presence of a Cl substituent at the 7-, 6- or 4-position of the benzothiazolyl moiety facilitates an orientation which enables complex stabilization via pi-interactions and hydrogen bond formation involving either the CO group of the thiazolidinone ring or hydrogen acceptor/donor substituents of the phenyl ring. In case of subgroups bearing hydrogen acceptor substituents at the benzothiazole moiety, their involvement in hydrogen bond formation also is favorable for complex stabilization.

The absence of the traditional hydrogen bond interactions with Lys101, in case of one of the most active compounds, **10**, is an interesting characteristic in concern of a probable use of such structures in future drugs, since mutations in Lys101 are common in resistant mutants.

Finally, it should be mentioned that no statistically significant changes were observed in cell growth in normal cell line treated with the tested compounds compared to control.

## Figures and Tables

**Table 1:**
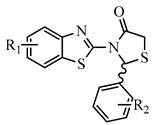
Molecular docking scores and PASS prediction of all the designed compounds.

N	R_1_	R_2_	Free Binding Energy (kcal/mol) S-(−)	Free Binding Energy (kcal/mol) R-(+)	Pa	Ν	R_1_	R_2_	Free Binding Energy (kcal/mol) -(−)	Free Binding Energy (kcal/mol) R-(+)	Pa
**a1 (1)**	7-Cl	2,6-di-F	−10.21	−13.37	0.651	**i7**	6-Br	2,3-di-Cl	−3,25	−4.07	0.352
**a2 (2)**	7-Cl	2-F, 6-Cl	−11.42	−14.10	0.704	**i8**	6-Br	2,4-di-Cl	−5,37	−6.43	0.341
**b1 (3)**	6-F	4-F	−10.95	−13.21	0.472	**k1**	6-CN	3-Cl	−5.29	−6.57	0.353
**b2 (4)**	6-F	4-NO_2_	−8.02	−9.11	0.374	**k2**	6-CN	3-Br	−4.18	−5.66	0.393
**b3 (5)**	6-F	4-Cl	−8.15	−9.02	0.398	**k3**	6-CN	3-F	−6.32	−703	0.287
**b4 (6)**	6-F	4-OCH_3_	−7.14	−8.70	0.360	**k4**	6-CN	2,6-di-Cl	−6.58	−7.84	0.498
**b5 (7)**	6-F	4-OH	−8.27	−9.27	0.415	**k5**	6-CN	2,3-di-Cl	−6.45	−7.16	0.447
**b6**	6-F	4-Br	−6.17	−6.54	0.401	**k6**	6-CN	2,4-di-Cl	−6.93	−7.18	0.366
**b7**	6-F	2,3-di-Cl	−6.53	−6.71	0.353	**l1**	6-CF_3_	2,6-di-Cl	−6.44	−7.12	0.448
**b8**	6-F	2,4-di-Cl	−6.05	−6.41	0.349	**l2**	6-CF_3_	3-Cl	−5.41	−6.27	0.332
**c1 (8)**	6-Cl	4-F	−10.13	−12.30	0.426	**l3**	6-CF_3_	3-Br	−5.07	−5.71	0.254
c2	6-Cl	2-Cl	−6.28	−6.85	0.372	**l4**	6-CF_3_	4-Br	−5.09	−5.82	0.378
c3	6-Cl	3-Cl	−6.55	−6.93	0.285	**l5**	6-CF_3_	2,3-di-Cl	−4.05	−4.70	0.336
c4	6-Cl	3-Br	−6.01	−7.11	0.402	**l6**	6-CF_3_	2,4-di-Cl	−6.41	−6.91	0.332
**c5 (9)**	6-Cl	4-OH	−11.08	−13.63	0.492	**m1**	6-Ad	3-Cl	−5.12	−6.74	0.273
**c6**	6-Cl	4-Br	−6.01	−7.11	0.419	**m2**	6-Ad	3-Br	−5.96	−6.89	0.280
**c7**	6-Cl	2,3-di-Cl	−6.25	−7.59	0.411	**m3**	6-Ad	2,6-di-F	−6.25	−7.04	0.428
**c8**	6-Cl	2,4-di-Cl	−5.92	−6.42	0.419	**m4**	6-Ad	2,3-di-Cl	−7.14	−8.05	0.289
**d1 (10)**	4-Cl	4-F	−11.16	−14.59	0.469	**m5**	6-Ad	2,4-di-Cl	−7.02	−7.94	0.285
**d2 (11)**	4-Cl	4-NO_2_	−8.45	−9.28	0.393	**m6**	6-Ad	4-F	−6.82	−7.88	0.325
**d3 (12)**	4-Cl	4-Cl	−8.73	−10.26	0.480	**m7**	6-Ad	4-NO_2_	−5.17	−6.75	0.226
**d4 (13)**	4-Cl	4-OCH_3_	−9.11	−11.69	0.378	**m8**	6-Ad	4-Cl	−6.79	−7.55	0.319
**d5 (14)**	4-Cl	4-OH	−10.58	−13.72	0.436	**m9**	6-Ad	4-OCH_3_	−6.49	−7.28	0.295
**d6**	4-Cl	4-Br	−6.17	−7.76	0.422	**m10**	6-Ad	4-OH	−4.18	−5.94	0.333
**d7**	4-Cl	2,3-di-Cl	−6.63	−7.15	0.424	**n1**	4-CH_3_, 6-Ad	3-Cl	−4.26	−5.97	0.226
**d8**	4-Cl	2,4-di-Cl	−7.14	−7.93	0.404	**n2**	4-CH_3_, 6-Ad	3-Br	−4.13	−5.62	0.280
**e1 (15)**	4-OCH_3_	4-F	−9.93	−11.51	0.353	**n3**	4-CH_3_, 6-Ad	2-F, 6-Cl	−7.03	−8.12	0.507
**e2 (16)**	4-OCH_3_	4-NO_2_	−10.05	−11.58	0.374	**n4**	4-CH_3_, 6-Ad	2,3-di-Cl	−6.58	−7.89	0.280
**e3 (17)**	4-OCH_3_	4-Cl	−9.92	−11.23	0.347	**n5**	4-CH_3_, 6-Ad	2,4-di-Cl	−6.93	−7.91	0.276
**e4 (18)**	4-OCH_3_	4-OCH_3_	−8.76	−10.97	0.380	**n6**	4-CH_3_, 6-Ad	4-F	−6.74	−7.59	0.313
**e5 (19)**	4-OCH_3_	4-OH	−10.15	−11.02	0.379	**n7**	4-CH_3_, 6-Ad	4-NO_2_	−5.08	−6.65	0.202
**e6**	4-OCH_3_	4-Br	−6.58	−7.46	0.329	**n8**	4-CH_3_, 6-Ad	4-Cl	−6.83	−7.94	0.307
**e7**	4-OCH_3_	2,3-di-Cl	−5.14	−6.03	0.317	**n9**	4-CH_3_, 6-Ad	4-OCH_3_	−6.37	−7.17	0.287
**e8**	4-OCH_3_	2,4-di-Cl	−5.79	−6.48	0.322	**n10**	4-CH_3_, 6-Ad	4-OH	−4.16	−5.93	0.321
**f1 (20)**	6-OCH_3_	4-F	−10.87	−12.17	0.393	**o1**	5,6-di-CH_3_	4-F	−6.95	−7.56	0.433
**f2 (21)**	6-OCH_3_	4-NO_2_	−7.14	−8.96	0.346	**o2**	5,6-di-CH_3_	4-NO_2_	−5.18	−6.05	0.328
**f3 (22)**	6-OCH_3_	4-Cl	−9.88	−11.00	0.365	**o3**	5,6-di-CH_3_	4-Cl	−5.21	−6.17	0.432
**f4 (23)**	6-OCH_3_	4-OCH_3_	−9.91	−11.49	0.420	**o4**	5,6-di-CH_3_	4-OCH_3_	−3.28	−4.56	0.327
f5	6-OCH_3_	3-Br	−5.03	−6.11	0.302	**o5**	5,6-di-CH_3_	4-OH	−4.19	−5.13	0.351
**f6**	6-OCH_3_	4-Br	−5.17	−6.13	0.367	**o6**	5,6-di-CH_3_	4-Br	−3.05	−4.27	0.355
**f7**	6-OCH_3_	2,3-di-Cl	−5.61	−6.92	0.303	**o7**	5,6-di-CH_3_	2,3-di-Cl	−6.17	−7.01	0.275
**f8**	6-OCH_3_	2,4-di-Cl	−6.24	−7.32	0.325	**o8**	5,6-di-CH_3_	2,4-di-Cl	−6.14	−6.86	0.286
**g1 (24)**	6-OCH_2_CH_3_	4-F	−6.97	−8.35	0.373	**p1**	6-NO_2_	4-F	−6.84	−6.92	0.390
**g2 (25)**	6-OCH_2_CH_3_	4-NO_2_	−7.14	−9.02	0.339	**p2**	6-NO_2_	4-NO_2_	−5.11	−6.01	0.225
**g3 (26)**	6-OCH_2_CH_3_	4-Cl	−9.84	−11.35	0.376	**p3**	6-NO_2_	4-Cl	−5.20	−6.15	0.382
**g4 (27)**	6-OCH_2_CH_3_	4-OCH_3_	−9.10	−10.98	0.356	**p4**	6-NO_2_	4-OCH_3_	−3.15	−4.42	0.348
**g5 (28)**	6-OCH_2_CH_3_	4-OH	−10.05	−11.56	0.387	**p5**	6-NO_2_	4-OH	−3.47	−4.18	0.398
**g6**	6-OCH_2_CH_3_	4-Br	−4.28	−5.43	0.358	**p6**	6-NO_2_	4-Br	−3.01	−4.15	0.284
**g7**	6-OCH_2_CH_3_	2,3-di-Cl	−5.84	−6.47	0.286	**p7**	6-NO_2_	2,3-di-Cl	−6.18	−6.81	0.341
**g8**	6-OCH_2_CH_3_	2,4-di-Cl	−5.33	−6.21	0.320	**p8**	6-NO_2_	2,4-di-Cl	−6.29	−6.98	0.337
**h1 (29)**	6-OCF_3_	2,6-di-Cl	−9.87	−11.03	0.388	**q1**	4-CH_3_	4-F	−7.01	−7.82	0.345
h2	6-OCF_3_	2,6-di-F	−7.77	−8.85	0.424	**q2**	4-CH_3_	4-NO_2_	−5.10	−6.12	0.326
h3	6-OCF_3_	3-Cl	−5.96	−6.88	0.315	**q3**	4-CH_3_	4-Cl	−5.03	−5.85	0.335
**h4 (30)**	6-OCF_3_	2,3-di-Cl	−7.23	−9.31	0.300	**q4**	4-CH_3_	4-OCH_3_	−4.85	−5.16	0.290
h5	6-OCF_3_	3-Br	−5.17	−6.33	0.242	**q5**	4-CH_3_	4-OH	−4.08	−5.49	0.353
h6	6-OCF_3_	4-F	−6.94	−8.74	0.345	**q6**	4-CH_3_	4-Br	−3.17	−4.55	0.338
**h7 (31)**	6-OCF_3_	4-NO_2_	−8.45	−9.07	0.315	**q7**	4-CH_3_	2,3-di-Cl	−6.14	−6.80	0.390
**h8 (32)**	6-OCF_3_	4-Cl	−6.89	−8.71	0.332	**q8**	4-CH_3_	2,4-di-Cl	−6.33	−6.86	0.382
h9	6-OCF_3_	3-F	−5.22	−6.19	0.330	**r1**	6-CH_3_	4-F	−7.00	−7.81	0.337
h10	6-OCF_3_	4-OH	−7.97	−8.51	0.442	**r2**	6-CH_3_	4-NO_2_	−5.02	−5.93	0.315
**i1**	6-Br	4-F	−7.31	−8.10	0.301	**r3**	6-CH_3_	4-Cl	−5.01	−5.83	0.327
**i2**	6-Br	4-NO_2_	−6.88	−7.95	0.326	**r4**	6-CH_3_	4-OCH_3_	−4.77	−5.12	0.284
**i3**	6-Br	4-Cl	−7.19	−8.02	0.393	**r5**	6-CH_3_	4-OH	−4.05	−5.40	0.345
**i4**	6-Br	4-OCH_3_	−5.35	−6.42	0.285	**r6**	6-CH_3_	4-Br	−3.12	−4.52	0.330
**i5**	6-Br	4-OH	−7.22	−8.02	0.409	**r7**	6-CH_3_	2,3-di-Cl	−6.13	−6.78	0.377
**i6**	6-Br	4-Br	−5.11	−5.84	0.405	**r8**	6-CH_3_	2,4-di-Cl	−6.10	−6.64	0.372
**Etravirine**			−11.25			**Nevirapine**			−11.95		

**Table 2:** Molecular docking scores and PASS prediction of the selected compounds.

N	Predicted Anti-HIV Activity (Pa)	Free Binding Energy (kcal/mol) S-(−)	Free Binding Energy (kcal/mol) R-(+)	H Bonds	Amino Acids	Hydrophobic Interactions	Pi Interact.	Halogen Interact.
**1**	0.651	−10.21	−13.37	1	Lys101	Thr139, Lys172, Gly190	Pro95, Leu100, Lys103, Glu138, Val179, Tyr181	Ile180
**2**	0.704	−11.42	−14.10	1	Lys101	Glu28, Gly99, Leu100, Lys103, Asn136, Tyr181, Ile382, Trp383	Glu138, Ile135, Val179, Tyr319, Pro321	Glu138
**3**	0.472	−10.95	−13.21	2	Lys101 Trp229	Val106, Gly190, Leu234	Leu100, Lys103, Glu138, Val179, Tyr181, Tyr188, Trp229, Leu234	-
**4**	0.374	−8.02	−9.11	1	Lys101	Glu131, Thr132	Leu100, Val106, Glu138	-
**5**	0.398	−8.15	−9.02	-	-	Glu138, Tyr181, Tyr188, Gly190, Phe227, Tyr318	Leu100, Lys101, Val106, Val179	Leu234 Pro236
**6**	0.360	−7.14	−8.70	1	Lys103	Ser127, Glu131	Leu100, Glu138, Val179, Tyr181	-
**7**	0.415	−8.27	−9.72	-	-	Pro95, Lys101, Lys103, Tyre181, Gly190, Phe117, Trp229, His235, Pro236, Tyr318	Leu100, Val106, Glu138, Val179, Tyr188, Leu234	Glu138
**8**	0.426	−10.13	−12.30	2	Lys101 Pro126	Glu23, Ser127, Glu131, Thr132, Pro176, Ile178, Ile180	Ile128, Arg172, Val179	-
**9**	0.492	−11.08	−14.63	2	Lys101 Thr139	Pro95, Lys103, Lys172, Tyr181, Leu234	Leu100, Glu138, Val179	-
**10**	0.469	−11.16	−14.59	1	Tyr318	Ile180, Tur181, Gly190, Trp229	Leu100, Lys103, Val106, Glu138, Val179, Tyr188, Leu234	Lys101 His235 Pro236
**11**	0.393	−8.45	−9.28	1	Lys101	Lys103, Val106, Ile180	Leu100, Val179, Tyr181	-
**12**	0.480	−8.73	−10.26	1	Lys101	Lys103, Ile180	Leu100, Glu138, Val179	
**13**	0.378	−9.11	−11.69	2	Lys101 Thr139	Lys103, Lys172, Ile180	Leu100, Glu138, Val179, Tyr181	-
**14**	0.436	−10.58	−13.72	3	Lys101 Glu138 Thr139	Val106, Leu234	Leu100, Lys103, Val179, Tyr181	-
**15**	0.353	−9.93	−11.51	2	Lys101 Trp229	Pro95, Val106, Tyr188, Leu234	Leu100, Lys103, Glu138, Val179, Tyr181	-
**16**	0.374	−10.05	−11.58	2	Lys101	Pro95, Val106, Tyr188	Leu100, Glu138, Val179, Tyr181	-
**17**	0.347	−9.92	−11.23	1	Lys101	Pro95, Ile180, Tyr181, Gly190, Trp229, His235	Leu100, Lys103, Glu138, Val179, Tyr188, Tyr318	-
**18**	0.380	−8.76	−10.97	2	Glu138 Tyr318	Pro95, Lys101, Lys102, Tyr181, Tyr188, Gly190, Trp229, His235	Leu100, Val106, Glu138, Val179, Leu234	-
**19**	0.379	−10.15	−11.02	1	Lys101	Lys103, Ile180, Tyr181, Gly190, Trp229, Pro236, Tyr318, His235	Leu100, Val106, Glu138, Val179, Tyr188, Leu234	-
**20**	0.393	10.87	−12.17	2	Lys101 Thr139	Pro95, Leu100, Lys103, Lys172, Ile180, Tyr188	Glu138, Val179, Tyr181, Trp229	-
**21**	0.346	−7.14	−8.96	1	Lys101	Pro95, Ile180, Tyr188	Leu100, Glu138, Val179	-
**22**	0.365	−9.88	−11.00	2	Lys101 Thr132	Pro95, Lys172, Ile180, Tyr188	Leu100, Glu138, Val179	-
**23**	0.420	−9.91	−11.49	2	Lys101 Tyr181	Lys102, Lys103, Ile180, Tyr188, Phe225, Pro234	Leu100, Val106, Val179, Trp227, Leu232, Tyr316	-
**24**	0.373	−6.97	−8.35	1	Lys101	Pro95, Lys103, Ile180, Tyr181	Leu100, Glu138, Val179	-
**25**	0.339	−7.14	−9.02	1	Lys101	Pro95, Gly99, Thr139, Ile180, Tyr181	Leu100, Glu138, Val179	-
**26**	0.376	−9.84	−11.35	1	Lys101	Pro95, Gly99, Lys103, Thr139, Lys172, Ile180, Tyr181	Leu100, Glu138, Val179, Trp229, Leu234	-
**27**	0.356	−9.10	−10.98	1	Lys101	Pro95, Pro97, Lys103, Thr139, Lys172, Ile180, Tyr181	Leu100, Glu138, Val179, Trp229, Leu234	-
**28**	0.387	−10.05	−11.56	2	Lys101 Thr139	Pro95, Pro97, Lys103, Lys172, Ile180	Leu100, Glu138, Val179, Trp229, Leu234	-
**29**	0.388	−9.87	−11.03	1	Lys101	Pro95, Tyr181, Gly190, Phe227, Pro236, Tyr318	Leu100, Lys103, Val106, Val179, Tyr188, Leu234	Glu138 Gly99
**30**	0.300	−7.23	−9.31	1	Lys101	Lys101, Tyr181, Gly190, His235	Leu100, Val179, Tyr188	-
**31**	0.315	−8.45	−9.07	1	Lys101	Pro95, Lys103, Lys172, Leu234	Leu100, Glu138, Val179,	-
**32**	0.332	−6.89	−8.71	2	Lys103 Thr132	Pro95, Tyr181	Glu138, Val179, Pro321	-
	**Nevirapine**	−11.95		2	Lys101, Glu138	Glu28, Gly99, Leu100, Lys101, Lys103, Val179, Ile382	Lys101, Glu138, Ile135, Tyr319, Trp383	-
	**Etravirine**	−11.25		2	Lys101, Glu138	Lys102, Val108, Ty188, Pro225, Phe227, Pro236	Pro95, Leu100, Lys103, Val106, Val179, Tyr181, Thr229, Leu234	-

**Table 3:** Predicted toxicity with program PROTOX.

Έ.	Predicted LD50	Predicted Toxicity Class	Hepatotoxicity	Carcinogenicity	Immunotoxicity	Mutagenicity	Cytotoxicity
**1**	**500 mg/kg**	4	Inactive 0.56	Inactive 0.57	Inactive 0.94	Inactive 0.67	Inactive 0.76
**2**	**500 mg/kg**	4	Inactive 0.56	Inactive 0.57	Inactive 0.95	Inactive 0.67	Inactive 0.76
**3**	**500 mg/kg**	4	Inactive 0.57	Inactive 0.55	Inactive 0.99	Inactive 0.66	Inactive 0.80
**4**	**1000 mg/kg**	4	Inactive 0.55	Inactive 0.68	Inactive 0.99	Inactive 0.66	Inactive 0.73
**5**	**500 mg/kg**	4	Inactive 0.57	Inactive 0.55	Inactive 0.99	Inactive 0.67	Inactive 0.81
**6**	**500 mg/kg**	4	Inactive 0.60	Inactive 0.58	Inactive 0.98	Inactive 0.65	Inactive 0.66
**7**	**500 mg/kg**	4	Inactive 0.63	Inactive 0.59	Inactive 0.99	Inactive 0.66	Inactive 0.70
**8**	**500 mg/kg**	4	Inactive 0.57	Inactive 0.55	Inactive 0.99	Inactive 0.67	Inactive 0.81
**9**	**500 mg/kg**	4	Inactive 0.60	Inactive 0.58	Inactive 0.99	Inactive 0.68	Inactive 0.68
**10**	**500 mg/kg**	4	Inactive 0.57	Inactive 0.55	Inactive 0.99	Inactive 0.67	Inactive 0.81
**11**	**1000 mg/kg**	4	Inactive 0.52	Inactive 0.65	Inactive 0.99	**Active 0.91**	Inactive 0.74
**12**	**500 mg/kg**	4	Inactive 0.54	Inactive 0.55	Inactive 0.99	Inactive 0.68	Inactive 0.82
**13**	**500 mg/kg**	4	Inactive 0.57	Inactive 0.58	Inactive 0.99	Inactive 0.66	Inactive 0.64
**14**	**500 mg/kg**	4	Inactive 0.60	Inactive 0.58	Inactive 0.99	Inactive 0.68	Inactive 0.68
**15**	**500 mg/kg**	4	Inactive 0.59	Inactive 0.60	Inactive 0.98	Inactive 0.66	Inactive 0.66
**16**	**1000 mg/kg**	4	Inactive 0.52	Inactive 0.65	Inactive 0.99	Inactive 0.66	Inactive 0.74
**17**	**500 mg/kg**	4	Inactive 0.56	Inactive 0.60	Inactive 0.98	Inactive 0.67	Inactive 0.65
**18**	**500 mg/kg**	4	Inactive 0.50	Inactive 0.62	Inactive 0.98	Inactive 0.61	Inactive 0.63
**19**	**500 mg/kg**	4	Inactive 0.52	Inactive 0.64	Inactive 0.99	Inactive 0.60	Inactive 0.63
**20**	**500 mg/kg**	4	Inactive 0.59	Inactive 0.60	Inactive 0.98	Inactive 0.66	Inactive 0.66
**21**	**1000 mg/kg**	4	Inactive 0.52	Inactive 0.65	Inactive 0.99	Inactive 0.66	Inactive 0.74
**22**	**500 mg/kg**	4	Inactive 0.56	Inactive 0.60	Inactive 0.98	Inactive 0.67	Inactive 0.65
**23**	**500 mg/kg**	4	Inactive 0.50	Inactive 0.62	Inactive 0.98	Inactive 0.61	Inactive 0.63
**24**	**500 mg/kg**	4	Inactive 0.52	Inactive 0.57	Inactive 0.96	Inactive 0.64	Inactive 0.62
**25**	**1000 mg/kg**	4	Inactive 0.53	Inactive 0.63	Inactive 0.97	Inactive 0.66	Inactive 0.62
**26**	**500 mg/kg**	4	Inactive 0.52	Inactive 0.56	Inactive 0.98	Inactive 0.65	Inactive 0.59
**27**	**500 mg/kg**	4	Inactive 0.53	Inactive 0.62	Inactive 0.97	Inactive 0.61	Inactive 0.61
**28**	**500 mg/kg**	4	Inactive 0.50	Inactive 0.62	Inactive 0.99	Inactive 0.63	Inactive 0.63
**29**	**500 mg/kg**	4	Inactive 0.61	Inactive 0.56	Inactive 0.91	Inactive 0.66	Inactive 0.64
**30**	**500 mg/kg**	4	Inactive 0.61	Inactive 0.56	Inactive 0.91	Inactive 0.66	Inactive 0.64
**31**	**1000 mg/kg**	4	Inactive 0.56	Inactive 0.64	Inactive 0.81	Inactive 0.66	Inactive 0.64
**32**	**500 mg/kg**	4	Inactive 0.61	Inactive 0.56	Inactive 0.97	Inactive 0.66	Inactive 0.65

**Table 4:** HIV-1 RT inhibitory action.

N	Inhibition % (4 μM)	IC_50_ (μM)	N	Inhibition % (4 μM)	IC_50_ (μM)	N	Inhibition % (4 μM)	IC_50_ (μM)
**1**	70	0.21	**12**	50	4	**23**	73	2.39
**2**	72	0.0047	**13**	65	1.99	**24**	36	>4
**3**	72	0.15	**14**	83	0.01	**25**	44	>4
**4**	54	3.8	**15**	69	2.08	**26**	73	2.48
**5**	44	>4	**16**	68	2.20	**27**	76	1.93
**6**	35	>4	**17**	67	2.60	**28**	62	2.00
**7**	50	4	**18**	72	2.69	**29**	49	4.28
**8**	83	0.26	**19**	71	2.62	**30**	46	>4
**9**	56	0.001	**20**	55	1.51	**31**	46	>4
**10**	63	0.001	**21**	50	4	**32**	46	>4
**11**	40	>4	**22**	72	2.76	Nevirapine		0.3
